# Identification of binding sites for ivacaftor on the cystic fibrosis transmembrane conductance regulator

**DOI:** 10.1016/j.isci.2021.102542

**Published:** 2021-05-15

**Authors:** Onofrio Laselva, Zafar Qureshi, Zhi-Wei Zeng, Evgeniy V. Petrotchenko, Mohabir Ramjeesingh, C. Michael Hamilton, Ling-Jun Huan, Christoph H. Borchers, Régis Pomès, Robert Young, Christine E. Bear

**Affiliations:** 1 Programme in Molecular Medicine, Hospital for Sick Children, 686 Bay Street, Toronto, ON M5G 0A4, Canada; 2 Department of Medical and Surgical Sciences, University of Foggia, Foggia, Italy; 3 Department of Chemistry, Simon Fraser University, Burnaby, Canada; 4 Segal Cancer Proteomics Center, Lady Davis Institute, Jewish General Hospital, McGill University, Montreal, Canada; 5 Center for Computational and Data-Intensive Science and Engineering, Skolkovo Institute of Science and Technology, Moscow 121205, Russia; 6 Gerald Bronfman Department of Oncology, Jewish General Hospital, McGill University, Montreal, Quebec H3T 1E2, Canada; 7 Department of Physiology, University of Toronto, Toronto, Canada; 8 Department of Biochemistry, University of Toronto, Toronto, Canada

**Keywords:** Biochemistry, Biological sciences, Biophysics, Medicine, Structural biology

## Abstract

Ivacaftor (VX-770) was the first cystic fibrosis transmembrane conductance regulator (CFTR) modulatory drug approved for the treatment of patients with cystic fibrosis. Electron cryomicroscopy (cryo-EM) studies of detergent-solubilized CFTR indicated that VX-770 bound to a site at the interface between solvent and a hinge region in the CFTR protein conferred by transmembrane (tm) helices: tm4, tm5, and tm8. We re-evaluated VX-770 binding to CFTR in biological membranes using photoactivatable VX-770 probes. One such probe covalently labeled CFTR at two sites as determined following trypsin digestion and analysis by tandem-mass spectrometry. One labeled peptide resides in the cytosolic loop 4 of CFTR and the other is located in tm8, proximal to the site identified by cryo-EM. Complementary data from functional and molecular dynamic simulation studies support a model, where VX-770 mediates potentiation via multiple sites in the CFTR protein.

## Highlights

• A photoactivatable probe of ivacaftor specifically modifies CFTR in membranes.
• The probe modifies CFTR at the fourth cytosolic loop (ICL4) and at a kink formed by tm8.
• Functional and molecular dynamic stimulation studies support ICL4 as a binding site.

## Graphical abstract

**Figure:**
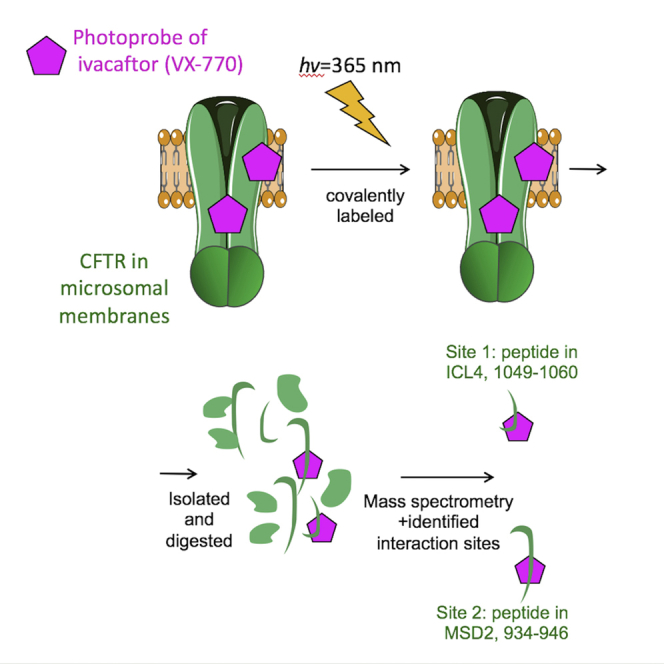

## Introduction

The cystic fibrosis transmembrane conductance regulator *(CFTR)* gene is expressed in multiple organs, including the lungs, pancreas, intestinal, and reproductive tract. Loss of function mutations in *CFTR* lead to cystic fibrosis disease, commonly associated with mucus obstruction of the airways, recurrent episodes of infection and progressive reduction of lung function. The discovery of modulatory compounds that partially rescue the protein defects conferred by CF-causing mutations promises to improve the health and well-being of most people with CF (Cuevas-Ocana et al., 2020).

The CFTR protein is an anion channel and a member of the ATP-binding cassette (ABC) superfamily of membrane proteins and shares the same multidomain architecture as related family members (Borst and Elferink, 2002). CFTR comprised two halves, with each half containing a membrane-spanning domain (MSD) linked to a nucleotide binding domain (NBD). The first half molecule containing MSD1 and NBD1 is connected to the second half, containing MSD2 and NBD2, via the R domain. PKA-mediated phosphorylation of the R domain is critical for its channel gating activity (Hwang and Sheppard, 2009; Kanelis et al., 2010; Ramjeesingh et al., 2008).

Substituted cysteine accessibility studies revealed that the anion selective pore of CFTR comprised multiple transmembrane-spanning helices, with the sixth helical segment playing a dominant role in conferring the selectivity filter (Gao et al., 2013; Negoda et al., 2019), According to biochemical and electrophysiological studies, PKA-dependent phosphorylation of CFTR in the R domain, promotes dissociation of the R domain from inhibitory interactions at the interface between the NBDs. This dissociation then enables ATP-mediated dimerization of the two NBDs (NBD1 and NBD2) (Kogan et al., 2002; Stratford et al., 2007; Vergani et al., 2005). Although, this NBD dimerized structure was associated with opening of the channel gate and chloride conduction through the pore in functional studies, the conformational events that lead to channel opening remain poorly understood. Interestingly, the helical extensions, comprising ICL1 through ICL4, that connect the NBDs to the pore in the membrane domain have been shown to contribute to CFTR channel gating (Hwang et al., 2018).

Electron cryomicroscopy (cryo-EM) structural models of CFTR confirmed its expected 3D architecture (Liu et al., 2017; Zhang et al., 2018). Interestingly, there were novel structural features revealed in these structures including, the lasso region (Liu et al., 2017; Zhang et al., 2018). The lasso is formed by the amino terminal region of CFTR and wraps around to interact with residues in MSD1 and MSD2. Importantly, a discontinuity in the eighth helical segment (tm8) of the second membrane-spanning domain was also revealed in the structures. The authors proposed that the hinge-like conformation in this region is important for channel gating (Liu et al., 2017; Zhang et al., 2018)

The small molecule, ivacaftor (VX-770), is a highly effective drug approved for those mutations that lead to reduced channel activity (Guerra et al., 2020; Van Goor et al., 2009, 2014). VX-770 has been shown to potentiate the channel activity of purified CFTR, suggesting that it acts by binding directly to CFTR (Eckford et al., 2012; Laselva et al., 2016). We and others showed that VX-770 augments CFTR channel open probability by stabilizing the open conductance state in the phosphorylated protein (Cui et al., 2019; Eckford et al., 2012).

The binding site for VX-770 on CFTR was examined by cryo-EM (Liu et al., 2019). In the structure of PKA-phosphorylated and ATP-bound human CFTR, VX-770 was localized at the putative interface between the hinge-like discontinuity in tm4, tm5, and tm8 and the lipid bilayer. Interestingly, there were no structural differences between the bound and unbound structures observed. These results contradicted those reported in a previous biophysical study, where ivacaftor binding to CFTR was assessed by hydrogen/deuterium exchange (HDX) mass spectrometry. The authors observed protection from deuterium exchange for the intracellular loop 4 (ICL4) (Byrnes et al., 2018) and suggested that VX-770 may bind to this region. Hence, uncertainty remains regarding the molecular basis for potentiation of CFTR by ivacaftor.

Both studies of ivacaftor binding were conducted with CFTR protein solubilized with detergents. There is emerging evidence to suggest that the function of the CFTR protein is modulated by lipids. Membrane extraction of CFTR using amphipols, amphipathic polymers that preserve associated lipids, resulted in a purified CFTR protein preparation with higher intrinsic activity than detergent-solubilized CFTR (Chin et al., 2019; Hildebrandt et al., 2017; Yang et al., 2014). Furthermore, addition of phospholipid back to detergent-solubilized CFTR protein enhances its catalytic activity as an ATPase (Chin et al., 2019). Therefore, we reasoned that these putative ivacaftor binding sites revealed for detergent-solubilized CFTR should be re-evaluated for CFTR in its natural context of the phospholipid bilayer. In the current study, we employed two photoactivatable probe analogs of VX-770 to identify its binding site on CFTR in biological membranes.

## Results

### VX-770 photoaffinity labeling probes are functional and label full length Wt-CFTR expressed in cell membranes

**Figure 1:**
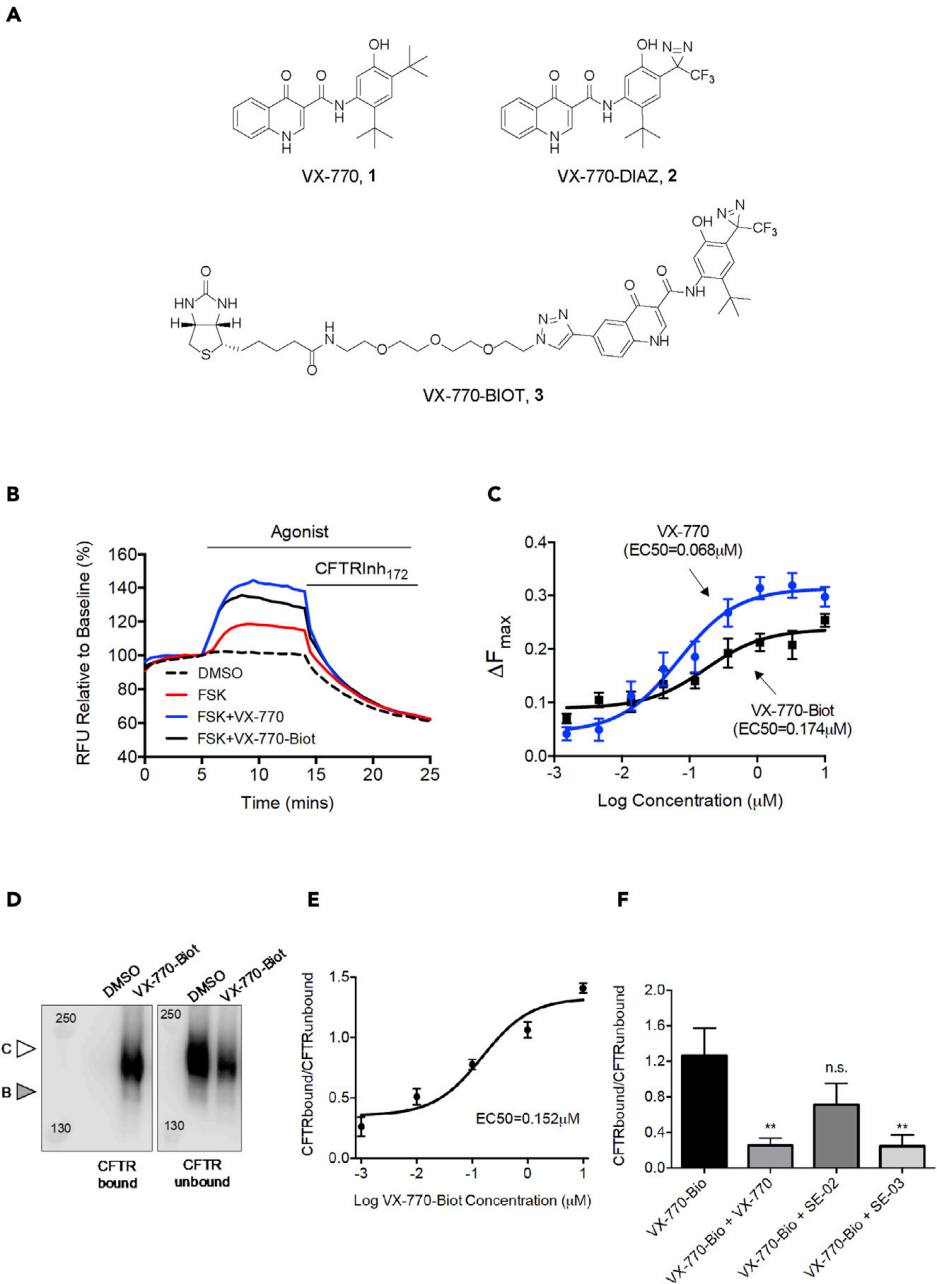
VX-770 probe potentiates and photolabels WT-CFTR (A) Structures of ivacaftor (VX-770, 1) and photoaffinity labeling probes VX-770-DIAZ (2) featuring a diazirine, and VX-770-BIOT (3) incorporating a biotin reporter tag. (B) Representative traces of WT-CFTR dependent chloride efflux (membrane depolarization assay) in HEK293 cells treated with FSK (1μM) +/− VX-770 (1 μM) or VX-770-Biot (1 μM). (C) Dose-response of VX-770 or VX-770-Biot (0.001-3μM) + FSK (1 μM) (±S.E.M) in WT-CFTR HEK293 cells. (D) Immunoblots of steady-state expression of WT-CFTR photolabeled with DMSO or VX-770-Biot (0.1μM). CFTR bound: CFTR biotinylated; CFTR unbound: CFTR unbiotinylated; Band C: mature, complex glycosylated CFTR; Band B: immature, core-glycosylated CFTR. (E) Dose-response of VX-770-Biot on WT-CFTR biotinylated protein expression in HEK293 cells (n = 3). (F) Bar graphs show the mean (±S.E.M) of the ratio of CFTR bound/CFTR unbound after treatment with VX-770-Biot (0.1 μM) +/− VX-770 (10 μM), SE-02 (10 μM) or SE-03 (10 μM) (n = 3-5). (∗∗p < 0.01)

Two photoaffinity labeling probes were used in this study based on the structure of ivacaftor (VX-770, 1, Figure 1A). The synthesis and characterization of the photoaffinity labeling probe VX-770-DIAZ (2) was reported recently (Hamilton et al., 2018) and was used in this work for labeling and mass spectrometry studies with Wt-CFTR. Moreover, to facilitate biochemical studies we generated a VX-770 probe with biotin as a reporter tag (VX-770-BIOT, 3, Figure 1A and Supplementary Chemistry Methods).

We confirmed that VX-770-BIOT is functional as a potentiator for CFTR (Figure 1B) using the FLiPR assay, designed in these studies to measure changes in membrane potential resulting from CFTR-mediated chloride efflux. As for the parent compound, VX-770, VX-770-BIOT augmented forskolin activated membrane depolarization as expected for its activity as a potentiator. The dose dependence for CFTR potentiation by VX-770-BIOT is shown in Figure 1C. While the efficacy of the biotinylated analog is reduced relative to the parent compound (VX-770 Emax = 0.31; VX-770-BIOT Emax = 0.25), it still augments the activation by forskolin by approximately 100% at sub-micromolar concentrations.

UV irradiation of membrane vesicles containing Wt-CFTR in the presence of VX-770-BIOT led to its covalent modification. This modification to CFTR was detected by applying solubilized HEK-293 crude membranes to a biotin affinity column. CFTR labeled with VX-770-BIOT and captured on the monoavidin column was then eluted using excess avidin. As shown in panels D-F, VX-770-BIOT labeled CFTR in a dose dependent manner. Importantly, VX-770-BIOT labeling of CFTR was competed by pre-exposure to the parent compound (VX-770) and also by an analog that exhibits potentiator activity (SE-03, (Chin et al., 2018)). A chemical analog that exhibits weak potentiator activity (SE-02, (Chin et al., 2018)) was ineffective in significantly competing VX-770-BIOT labeling on the full-length Wt-CFTR protein. Together, these findings support the proposal that this photoprobe of VX-770 labels CFTR at the site(s) to which VX-770 binds to mediate its activity as a potentiator.

### Identification of the VX-770-DIAZ binding sites by mass spectrometry

**Figure 2:**
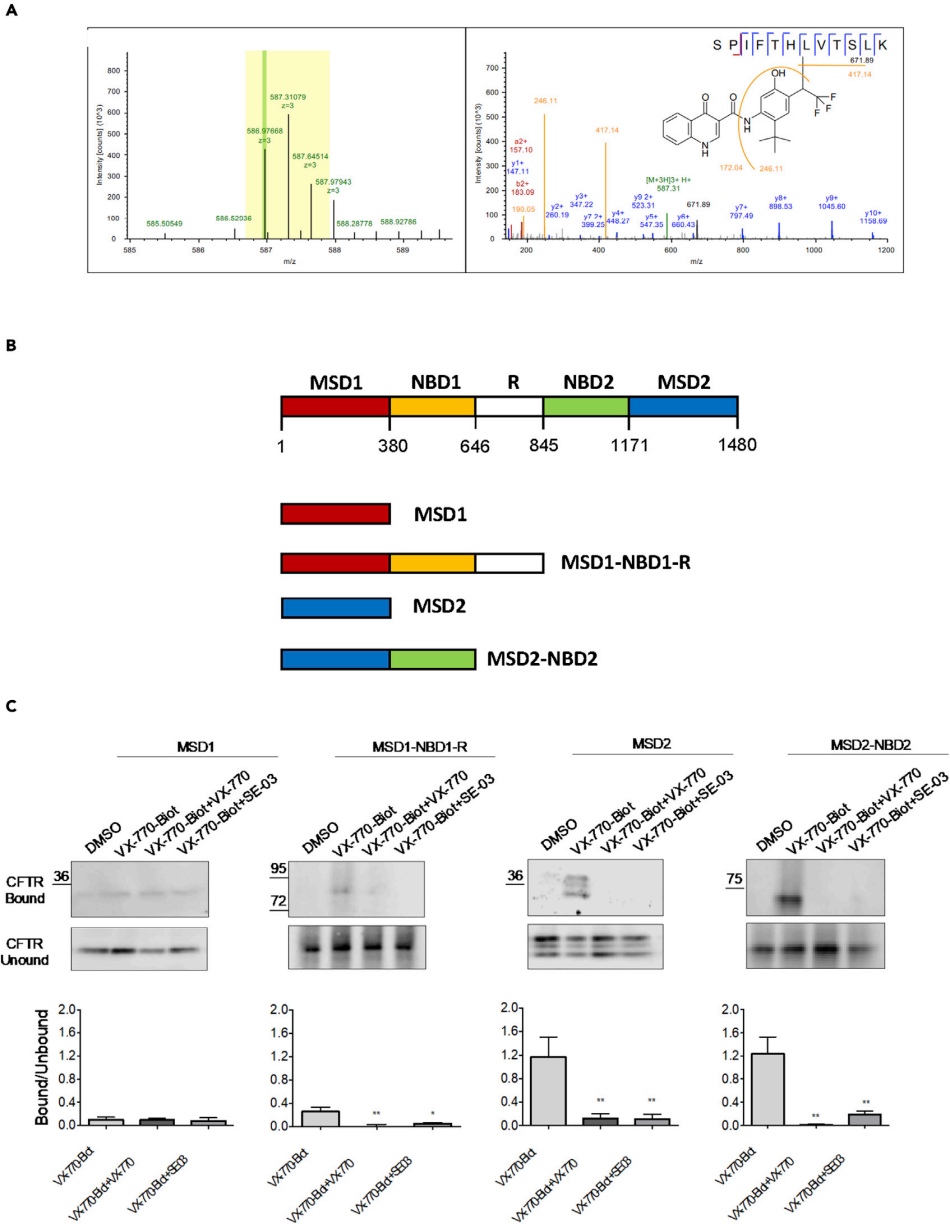
VX-770 probe photolabels full length Wt-CFTR protein on peptide (1049-1060) in MSD2 (A) After trypsin digestion of photolabeled CFTR, mass spectrometry studies were conducted to detect labeled residues. We show the spectra corresponding to the photolabeled peptide: 1049-1060. Left panel, MS1 spectrum of the 3 + precursor, monoisotopic mass is highlighted in green, m/z isolation window is highlighted in yellow. Right panel, MS2 fragmentation spectrum, diagnostic VX-770 reporter fragment ions are highlighted in orange. (B) In complementary studies, we assessed photolabeling of independent domains and we show the boundaries of these CFTR fragments. (C) Immunoblots of steady-state expression of WT-CFTR photolabeled with VX-770-Biot (0.1 μM) +/− VX-770 (10 μM) or SE-03 (10 μM). Bar graphs show the mean (±S.E.M) of the ratio of CFTR bound/CFTR unbound (n = 3-5). CFTR bound: CFTR biotinylated; CFTR unbound: CFTR unbiotinylated. (B) (∗p < 0.05, ∗∗p < 0.01)

In order to identify the residues that were modified by the VX-770 photolabel, we analyzed the full-length CFTR protein by mass spectrometry. First, the photoaffinity labeling reaction mixtures were separated by SDS-PAGE; regions of the gels corresponding to the molecular weight of CFTR were excised and digested (in-gel) with trypsin. The resulting peptides were analyzed by nanoscale liquid chromatography coupled to tandem mass spectrometry (nano-LC-MS) using Q-Exactive plus the Orbitrap mass spectrometer (Figure 2A). Data were searched against the human proteome database using variable modification settings corresponding to the non-selective covalent modification with VX-770-DIAZ probe 2. As a test set for the MS/MS analysis, bovine serum albumin (BSA) photo-labeled with VX-770-DIAZ and digested with trypsin was analyzed to identify modified peptide fragments specific to VX-770-DIAZ (Hamilton et al., 2018). Apparently, VX-770-DIAZ-modified peptides undergo facile MS/MS fragmentation at the bond connecting the VX-770 moiety with the peptide. This bond cleavage produces prominent characteristic peaks in MS/MS spectra, which can be used as diagnostic fragments that are specific for the VX-770-DIAZ-modified peptides. Unfortunately, this facile bond cleavage precludes detection of VX-770-containing b- or y-fragments and therefore prevents locating modified amino acid residues within the peptides. Using these reporter fragment ions, we have identified CFTR peptide: 1049-1060 in ICL4 as being modified by VX-770-DIAZ (Figure 2A). This modified peptide was reproducibly detected when photo-labeling of flash-frozen (Ziemianowicz et al., 2017) membrane material was performed. In the flash-frozen irradiated sample a weak signal corresponding to a second VX-770-DIAZ-modified peptide was also detected, in tm8 spanning residues 934-946 of CFTR.

CFTR fragments corresponding to isolated membrane-spanning domains (including extra- and intracellular loops that connect their intrinsic transmembrane helices) or membrane domain fusions with cytosolic domains, were expressed in HEK-293 cells and membrane vesicles were prepared from the transfected cells (Figure 2B). The VX-770-BIOT photoprobe was reacted with each of these membrane preparations in order to confirm the region that contains the binding site. As shown in Figure 2C, VX-770-BIOT labeling that could be competed with VX-770 and its active analog (SE-03), was observed predominantly on fragments containing MSD2. Minor labeling was observed for the fragment containing MSD1-NBD1 and the R domain. These labeling studies suggest that MSD2, which comprised 6 transmembrane helices and their connecting extramembrane loops, contains the site with which the photolabel is interacting. Together, these findings are compatible with the MS studies shown in Figure 2A and suggest that the VX-770 photoprobe interacts with MSD2 at a site in ICL4, previously identified by HDX (Byrnes et al., 2018), and at the binding site identified by cryo-EM that comprises tm4, tm5 and tm8 (Liu et al., 2019).

### Mutations targeting putative VX-770 binding sites in CFTR decrease its potentiation and interaction with photoactivatable probe

**Figure 3:**
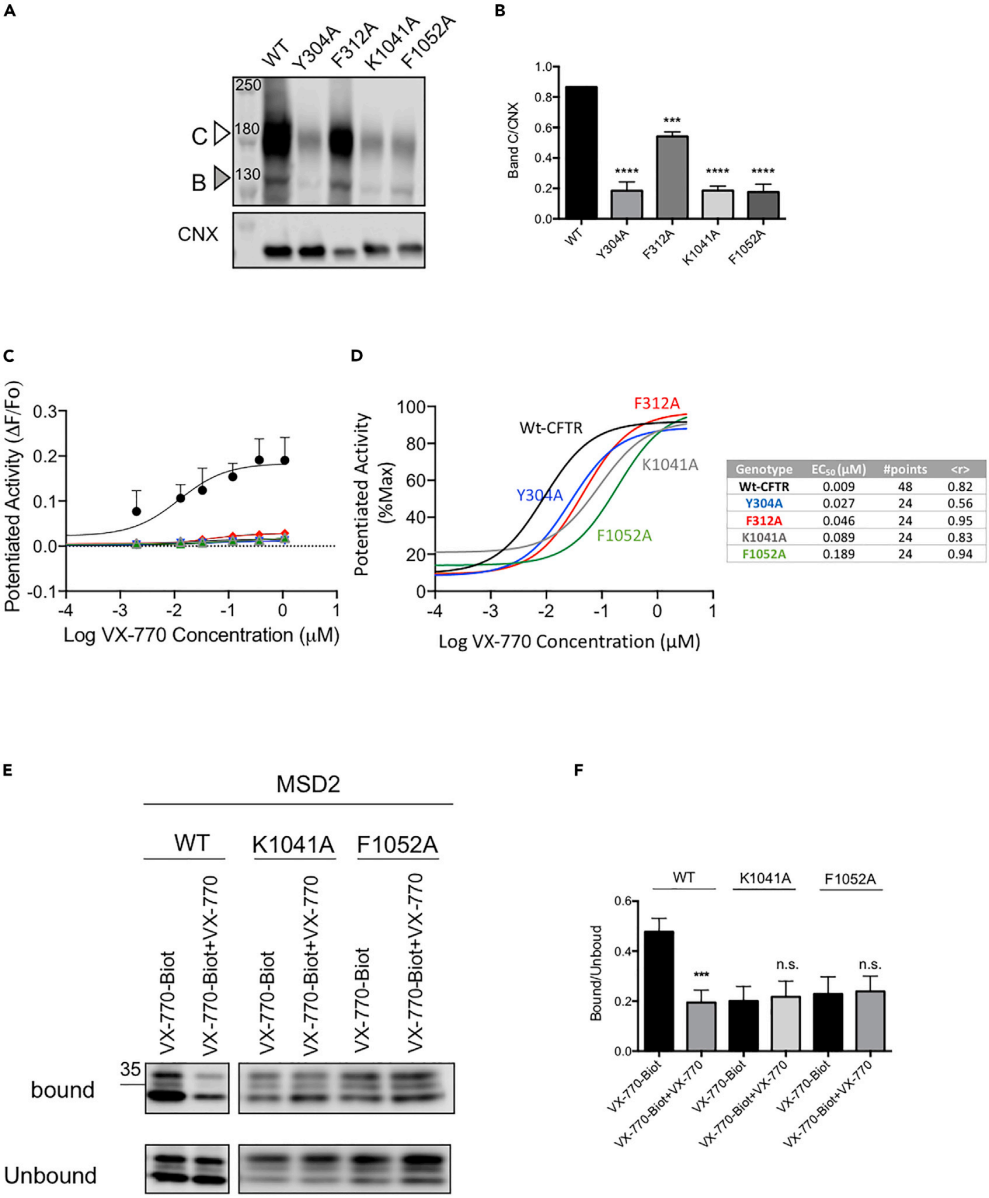
Mutations, K1041A and F1052A modify potency of VX-770 and reduce specific photolabeling of MSD2 (A) Immunoblots of steady-state expression of WT-, Y304A, F312A, K1041A, F1052A-CFTR in HEK293 cells. Band (C): mature, complex glycosylated CFTR; Band B: immature, core-glycosylated CFTR*,* CNX: calnexin. (B) Bar graphs show the mean (±S.E.M) of the ratio of Band C/CNX (n=3). (C) Dose-response of VX-770 (0.001-3 μM) + FSK (1 μM) (±S.E.M) in WT, Y304A, F312A, K1041A, F1052A-CFTR HEK293 cells (n = 3). (D) Dose-response of VX-770 normalized to the maximal response (n = 3). (E) Immunoblots of steady-state expression of MSD2, K1041A-MSD2, F1052A-MSD2 photolabeled with VX-770-Biot (0.1μM) +/− VX-770 (10μM). CFTR bound: CFTR biotinylated; CFTR unbound: CFTR unbiotinylated. (F) Bar graphs show the mean (±S.E.M) of the ratio of CFTR bound/CFTR unbound (n = 3). (∗∗∗p < 0.001, ∗∗∗∗p < 0.0001).

We generated single site mutations in order to compare the relative roles of the two regions in CFTR that were labeled using the photoactive probe, i.e., ICL4 and the site comprised by tm4, tm5, and tm8 in channel potentiation. The mutant CFTR proteins harboring K1041A or F1052A (Figure 3) were produced in order to assess the role of the labeled site in ICL4 in potentiation. The lysine (K) at position 1041 forms hydrogen bonds with residues in tm10 and tm11 and has been shown to be important for channel conduction (El Hiani and Linsdell, 2015). Mutation of the phenylalanine at position 1052 has been shown to reduce single channel open probability and the mutation, F1052V is associated with cystic fibrosis with varying clinical consequences (https://www.cftr2.org). The mutant CFTR proteins, Y304A and F312A, have been shown to alter VX-770 affinity to the VX-770 binding site identified by Liu et al. in cryo-EM studies (Liu et al., 2019).

As shown in Figures 3A and 3B, all of these mutant proteins were expressed as both the mature (complex glycosylated band C) and immature (core glycosylated band B) forms of the protein. However, the relative abundance of the mature protein form was reduced to a variable degree in each case (Figure 3B). These findings suggest that the assembly of the full length CFTR protein and its processing in the Golgi is compromised by these mutations. The efficacy for activation by the cAMP agonist, forskolin and potentiation by VX-770 is reduced for all of the mutant proteins relative to Wt-CFTR (Figure 3C). Importantly, this effect on macroscopic, CFTR-mediated channel function reflects, in part, the relatively low abundance of the plasma membrane localized form of mutant protein and/or its intrinsic activity as a phosphorylation and nucleotide regulated channel.

We assessed the consequences of the above mutations on the EC_50_ for VX-770 potentiation after normalization to their maximum activities (Figure 3D). These analyses suggested that mutations at the site containing Y304 and F312 conferred modest reductions in potency relative to those changes associated with the ICL4 mutants, i.e., K1041A and F1052A. These findings may be interpreted to suggest that VX-770 has a higher affinity to the ICL4 binding site than the site identified by Liu et al. (Liu et al., 2019). However, the functional expression of each of these mutants is low and there is a relationship between channel open probability and potentiation by VX-770 as defined by (Yeh et al., 2019). Hence, it is prudent to favor the conservative interpretation, that both sites are implicated in VX-770 binding and/or efficacy.

Because these functional studies do not allow us to unambiguously distinguish between their impact on VX-770 binding or potentiated channel gating, we were prompted to determine the relative contribution of photolabeling of the 1049-1060 helix to total labeling on MSD2 by VX-770-BIOT. In Figure 3E, we repeated the study showing that MSD2 is covalently labeled using VX-770-BIOT, and this labeling is competed with the parent compound, VX-770. Importantly, fractional labeling was reduced for MSD2 bearing either K1041A or F1052A relative to the Wt-MSD2. Further, the remaining photolabeling on either of these mutant domains was not competed by the parent compound (Figures 3E and 3F). These findings support the claim that ICL4 is a primary site for specific photolabeling by the VX-770 probe. Unfortunately, the low expression of full-length CFTR bearing site specific mutants prevented replication of the same studies in the context of the intact CFTR protein.

### Probing the CFTR:VX-770 binding sites by molecular dynamics simulations

The preceding results of biochemical and functional studies of full length CFTR and CFTR fragments point to the existence of a VX-770 binding site in ICL4 and the Liu site comprising TM4, TM5, and TM8 (Liu et al., 2019). In order to further test the relevance of these binding sites, we performed atomistic molecular dynamics (MD) simulations in the microsecond timescale on phosphorylated and ATP-bound human CFTR inserted into a POPC bilayer (pdb: 6MSM). We also employed an octanol slab as a model membrane in additional simulations in order to enhance sampling of potential VX-770 binding sites inside the transmembrane domain.

In order to define relatively high affinity binding sites, we employed a system that included one molecule of VX-770 added randomly to the aqueous solutions on both sides of a lipid bilayer containing one molecule of CFTR. By first approximation, the structural integrity of CFTR in our simulations was preserved according to root-mean-squared-deviation of Cα positions of membrane-spanning domains (Figure S1), and our simulations uncovered uncover potential binding sites and binding poses of VX-770 to CFTR. Our analysis of MD trajectories revealed 7 apparent binding sites for VX-770 (Figures S2–S4). To gauge the relative confidence for apparent binding in our simulations, we computed the potential energy of interaction for each identified binding event.

**Figure 4:**
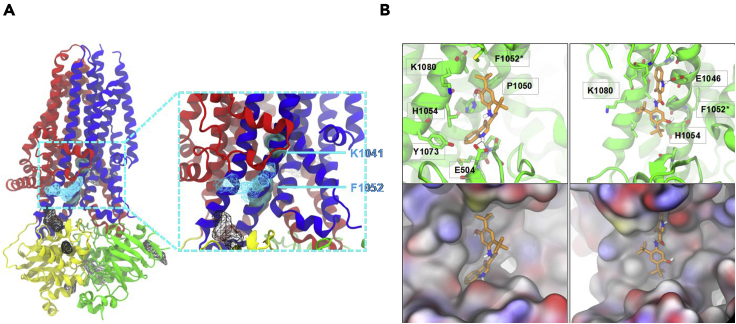
Molecular simulations showing that VX-770 potentially interacts at multiple sites on CFTR in a lipid bilayer (A) Overview of the structure of human CFTR (PDB: 6MSM) with VX-770 binding regions based on MD simulations shown in mesh representation. The ordered domains of CFTR are color coded (red: Lasso motif-MSD1; yellow: NBD1; blue: MSD2; green: NBD2). All mesh are shown in black except at photolabeling-MS identified location c which is shown in cyan. Two functional residues investigated through mutagenesis, K1041 and F1052, are shown in sphere representation as indicated. (B) Snapshots of VX-770 bound to a protein cavity at location c. On the top, stick representations of VX-770 and neighboring protein residues are shown (heavy atom contact distance cutoff: 0.45 nm). On the bottom, surface representations of the protein cavities are shown (red: oxygen; blue: nitrogen; yellow: sulfur; gray: carbon and hydrogen). The two binding poses shown correspond to the two most favorable binding events in Figure S2-A in terms of potential energy of interaction with CFTR.

The top two binding sites (labeled at sites a and b; Figures S2–S4), were located in NBD2 or at the interface between NBD1 and NBD2 respectively. These sites are unlikely to be required for potentiation by VX-770 since this activity does not require NBD2 (Jih and Hwang, 2013; Yeh et al., 2015). On the other hand, the next most energetically favorable location is consistent with our photolabeling studies, and it is situated in ICL4 (shown as the blue mesh, Figure 4A). This site was visited by three different simulation trajectories of CFTR both in POPC and in octanol (Figure 4, S2, and S2A).

**Figure 5:**
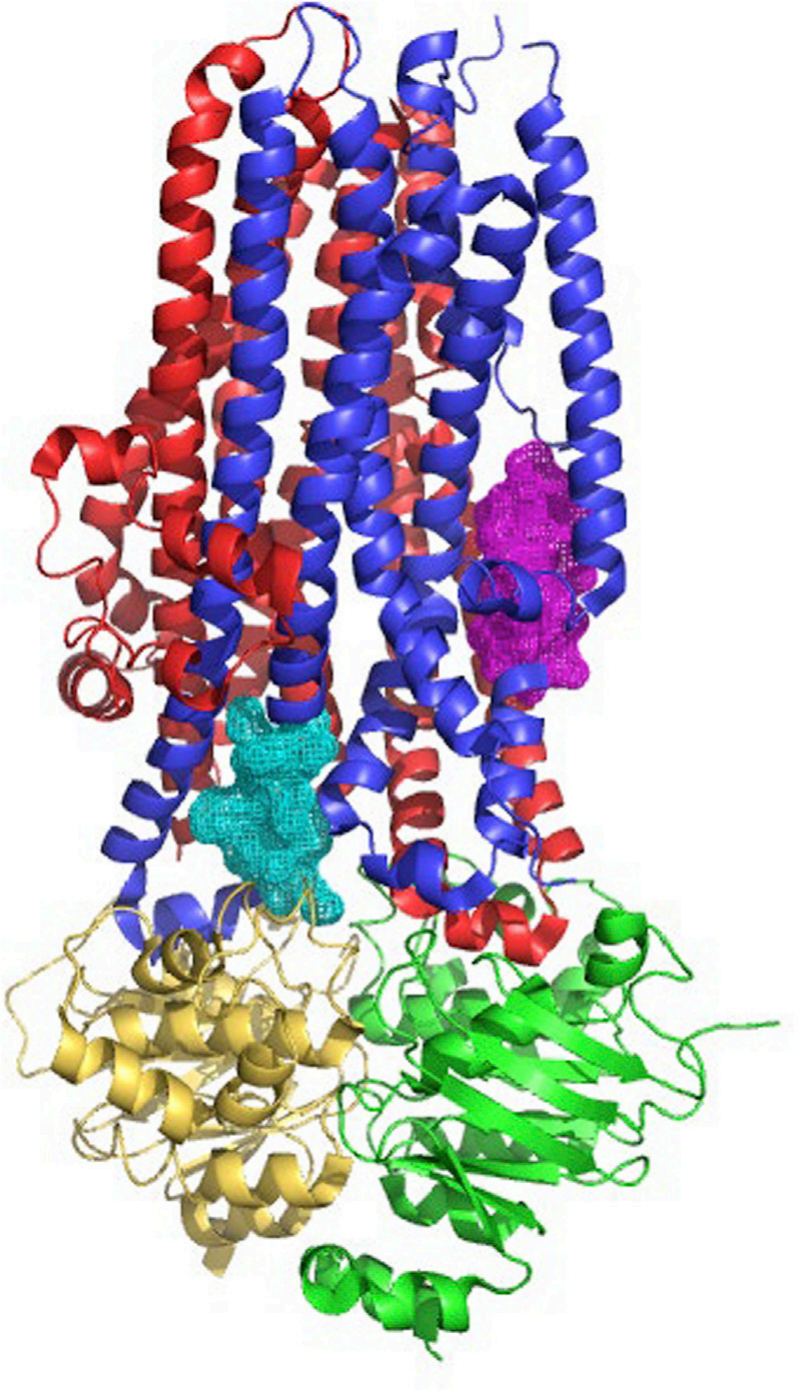
Molecular rendering showing the positions of two peptides that were photolabeled by VX-770 DIAZ The major domains of CFTR are colored as in Figure 4 with MSD1 in red, NBD1 in yellow, MSD2 in cobalt blue and NBD2 in green. The magenta mesh shows the labeled helix extending from 934-946 and the cyan mesh corresponds to labeled helix extending from 1049-1060. The cryo-EM studies by Liu et al. (Liu et al., 2019) identified VX-770 coordinated by residues 304, 308, 312, and 933, close to the position shown as the magenta mesh. The molecular dynamics simulations in the present study showed interaction of VX-770 with the region highlighted by the cyan mesh. Similarly, HDX studies by Byrne et al. (Byrnes et al., 2018) implicated the ICL4, proximal to the helix shown as cyan. Cartoon generated from pdb. 6MSM using PYMOL 2.4.1.

In both poses, extensive contacts were formed between VX-770 and residues of TM10 and TM11 helices flanking ICL4 (Figures 4 and 5, right cartoon). Both poses are consistent with the SAR previously described for VX-770 with intermolecular hydrogen bonding involving the quinoline nitrogen of VX-770 (Hadida et al., 2014). The pose in the left panel of Figure 4B also captures the critical hydrogen bond formation between the phenolic hydroxyl of VX-770 and the CFTR protein. The binding pose shown as a black mesh in Figure 4 (location *d* in Figures S2 and S3) is the fourth most energetically favorable pose and located close to the coupling helix conferred by ICL4 and spanning residues 1066-1076.

Interestingly, the site identified in cryo-EM structures (labeled as violet mesh in Figure 5, right cartoon) was not found in simulations that employed either POPC or octanol as the solvent. This negative finding supports our proposal that this region may constitute a relatively low affinity binding site or, alternatively, that longer simulations are required to detect binding to this site.

## Discussion

We identified a site using a novel photoactivatable probe, through which VX-770 may mediate its allosteric regulation of the CFTR chloride channel in biological membranes. Our evidence supports a model where VX-770 binds specifically to two sites, to a region on ICL4 that links the cytosolic NBD1 to the second membrane-spanning domain and to the region previously identified by cryo-EM, at the interface of membrane lipid and tm4, tm5, and tm8 of CFTR.

Photoactivatable probes have been used to identify drug binding sites in other clinically relevant membrane proteins (Ge et al., 2018; Hall et al., 2010). For example, binding sites for ligands for GABA_A_ receptors (Hall et al., 2010), GPCRs, the A2A receptor and NK1 receptor have recently been identified using the photoaffinity label approach. As in the current study, attached affinity (i.e., biotin) tags confirmed binding and facilitate isolation of labeled proteins (Muranaka et al., 2017; Muskens et al., 2019).

Photolabeling of ICL4 suggests that this region is a target for VX-770 binding and channel potentiation. This interpretation is consistent with previous electrophysiological studies and is in agreement with prior studies of CFTR using hydrogen-deuterium exchange (Byrnes et al., 2018). VX-770 has been shown to modulate ATP-independent gating of CFTR and the intracellular loops, including ICL4 have been implicated in this mode of ATP-independent, phosphorylation regulated gating (Cotten et al., 1996). In the putative open state of the CFTR channel, where the R domain is displaced from the core domains and the NBDs are dimerized, the intracellular loops 1 through 4, interact to form a tetra-helical bundle (Ge et al., 2018; Zhang et al., 2018). Wang and colleagues have identified interactions amongst the intracellular loops that promote channel opening (Wang et al., 2014). Typically, in these experiments, pairs of charged residues were found to regulate the stability of the channel open state. For example, mutation of K1060 to an alanine in ICL4 decreased the channel open time, presumably by disrupting the formation of a salt bridge with E267 proposed to stabilize the open conduction state. We speculate therefore that VX-770 binding to ICL4 may act to stabilize this salt bridge in order to facilitate the open channel structure of the phosphorylated, NBD dimerized CFTR protein.

Recently, we showed that VX-770 potentiated zebrafish CFTR (zCFTR) (Laselva et al., 2019). As expected, the site identified by cryo-EM in the human CFTR, including residues: F312 and Y304 and the residues in the novel site in ICL4, namely, K1041A and F1052A is conserved at corresponding positions in zCFTR. On the other hand, Bose et al. recently showed that VX-770 failed to potentiate murine Wt-CFTR (Bose et al., 2019). Further studies are required to fully understand why VX-770 is ineffective in potentiating murine CFTR, yet sequence alignment (UniProtKB/Swiss-Prot) showed that there are non-conservative amino acid substitutions in the coupling helix of ICL4. In addition, the tyrosine residue at position 304, constituting the binding site identified by Liu et al. (Liu et al., 2019) in hCFTR, is substituted with phenylalanine in the mouse polypeptide. Moreover, mutating the native tyrosine to phenylalanine in the mCFTR restores the potentiating effect of VX-770 (Yeh et al., 2019).

Multiple allosteric binding sites for modulatory ligands have been identified for GPCR membrane proteins (Muranaka et al., 2017; Muskens et al., 2019). In these cases, primary and secondary binding events have been shown to modulate long range conformational changes. Future studies are required to fully understand the mechanism through which VX-770 binding to ICL4 and the site involving the kink in tm8 potentiates CFTR channel activity. To date, such studies are limited by the lack of a structure of the open, chloride conducting CFTR channel. We anticipate that our finding of the role for ICL4 in VX-770 modified CFTR channel opening will drive further study of the network of long-range conformational changes required for opening and stabilization of the open channel gate.

Interestingly, our analyses of the effect of mutations at either site in full length CFTR revealed variable deleterious effects on protein maturation and processing (Figure 3E). At least for the limited number of mutations studied, reductions in protein processing efficiency did not appear to correlate with the relative reduction in VX-770 potency (Figure 3D). Nevertheless, these findings do highlight the potential of these mutations to perturb the intramolecular interactions critical for CFTR assembly and biosynthetic maturation. The potential impact of these mutations on intradomain folding and assembly was also apparent in studies of mutant MSD2 proteins (Figure 3E). We, and Clarke et al. (Laselva et al., 2018b; Loo et al., 2013), previously documented the multi-banding pattern observed for MSD2 (comprising both transmembrane helices, intracellular and extracellular loops). These are various glycosylation states of MSD2 and the relative abundance of the complex glycosylated form of MSD2 is modified by both mutations in MSD2 (Figure 3F). Clearly, single site mutations in MSD2 not only alter local structure but also change intradomain interactions and folding as reported by glycosylation in the Golgi. Hence, these biochemical findings highlight the impact of single site mutants on the tertiary structure of each domain and potentially, long range, intermolecular interactions.

In summary, our findings support the concept of a bivalent signaling network where independently, or in concert with its binding to transmembrane helix 8, VX-770 stabilizes the channel open configuration by binding to ICL4 in the tetrahelical bundle that connects the NBDs to the conduction path of the CFTR channel.

### Limitations of the study

We observed complex effects of introducing mutations targeting the probe binding sites, including changes in phosphorylation dependent gating and/or CFTR protein processing. These complex effects limited our ability to assign specific changes in ivacaftor efficacy related to site-directed mutation to disruption of its binding.

## STAR★methods

### Key resources table

**Table:** 

REAGENT or RESOURCE	SOURCE	IDENTIFIER
**Antibodies**		

Anti-CFTR	UNC CFTR antibodies	ID: 596
Anti-CFTR MM13-4	Merckmillipore	Cat#05-581, RRID:AB_309817
Anti-Calnexin	Sigma-Aldrich	Cat#C4731, RRID:AB_476845

**Chemicals**		

Forskolin	Sigma-Aldrich	Cat#F3917
VX-770 (Ivacaftor)	Selleck Chemicals	Cat#S1144
CFTRInh-172	Selleck Chemicals	Cat#S7139
VX-770-DIAZ	(Hamilton et al., 2018)	n/a
VX-770-BIOT	(Hamilton et al., 2018)	n/a

**Experimental models: Cell lines**		

Human Embryonic Kidney (HEK)-293 cells	Laboratory of Dr. Daniela Rotin	n/a

**Software and algorithms**		

ImageStudioLite	LI-COR Biosciences	https://www.licor.com/bio/image-studio-lite/download
GraphPad Prism	GraphPad	https://www.graphpad.com/

### Resource availability

#### Lead contact

Further information and requests should be directed to and will be fulfilled by the lead contact, Christine E. Bear, PhD (bear@sickkids.ca)

#### Materials availability

Materials (plasmids) generated in this study will be made available upon reasonable request to the lead contact.

#### Data and code availability

Topology and parameter files of VX-770 generated using GAAMP can be found on github (https://github.com/wilzzw).

### Method details

#### Cell culture and transfection

Human embryonic kidney (HEK) 293 GripTite™ cells (HEK293) (a gift from Dr. Daniela Rotin, Hospital for Sick Children, Toronto, Ontario, Canada) were maintained in DMEM (Wisent, St-Bruno, QC) supplemented with non-essential amino acids (Life Technologies, Waltham, MA) and 10% fetal bovine serum (FBS; Wisent, St-Bruno, QC) at 37 °C and processed with 5% CO_2_ as previously described (Laselva et al., 2021; Laselva et al., 2020b; Laselva et al., 2020c).

#### Generation of photoactivatable probes

Photoactivatable probes based on the structure of VX-770 were synthesized where the 4’-t-butyl group was replaced by a photoreactive trifluoromethyldiazirine group. The simple analog, VX-770-DIAZ (2) was synthesized as reported by Hamilton et al. (Hamilton et al., 2018) and an analog, VX-770-BIOT (3), bearing a biotin containing chain at the 6-position on the quinolone moiety and the 4’-trifluoromethyldiazirine, was synthesized as described herein (Supplementary Chemistry Methods). Both probes were prepared in protected forms for long term cold storage (where the phenolic group was blocked with an ethyloxymethyl group which was removed carefully as the last step before use. The protected forms of the probes were relatively stable when kept out of direct light (ambient and UV): however, once deprotected the probes were very sensitive to light, but could be stored stably in darkness at -80 ^o^C as either solutions in DMSO or as dry powder (stable at least 1 year).

#### Expression of full length Wt and mutant CFTR and CFTR fragments in HEK-293 cells, preparation of microsomes and photolabeling

Crude membranes were prepared from HEK-293 cells transiently expressing WT-CFTR or CFTR fragments: MSD1 (1-380) , MSD1-NBD1-R (1-845), MSD2 (837-11969), K1041A-MSD2, F1052A-MSD2 and MSD2-NBD2 (850-1480) and treated with 5mM NaBu for 24h at 37°C as previously described (Laselva et al., 2018a; Molinski et al., 2018). Briefly, cells pellets were resuspended in cell lysis buffer (10mM HEPES, 1mM EDTA, pH 7,2) and cells were lysed using a cell disruptor (10,000 psi, 4°C for 5 min). The cells suspension was centrifuged at 1900 rpm for 10 min at 4°C to pellet unbroken cells, and crude membranes were isolated from the resulting supernatant after centrifugation at 45,000 rpm for 60 min at 4°C. The crude membrane pellet was resuspended in buffer (40 mM Tris-HCl, 5 mM MgCl_2_, 0.1 mM EGTA, pH 7,4) by passage through a 1mL syringe 20 times with a 27-gauge needle (Molinski et al., 2017).

For photolabeling studies, 20μg crude membranes were treated with 0.1% DMSO, 0.1μM VX-770-Biot +/- 10μM VX-770, 10μM SE-02 or 10μM SE-03 (Chin et al., 2018). The samples were exposed to UV lamp for 15 min at 4°C and then resuspended with modified radioimmunoprecipitation assay (RIPA) buffer (50 mM Tris-HCl, 150 mM NaCl, 1 mM EDTA, pH 7.4, 0.2% SDS, and 0.1% Triton X-100) and centrifuged at 13,000 rpm for 10 min. The supernatant was then incubated with high capacity streptavidin agarose (Thermofisher) for 1h at room temperature. The biotinylated CFTR was than eluted by washing the streptavidin agarose with Elution buffers (Pierce Monomeric Avidin Agarose Kit, Thermofisher) and analyzed by SDS-PAGE on a 6% gel for WT-CFTR or a 4-12% gradient gel for CFTR fragments. After electrophoresis, proteins were transferred to nitrocellulose membranes (Bio-Rad, Hercules, CA) and incubated in 5% milk. CFTR bands were detected with human CFTR-NBD2-specific murine mAb 596 (1:5,000) or with human CFTR-MSD1-specific murine mAb MM13-4 (1:5,000). The blots were developed with ECL (Amersham) using the Li-Cor Odyssey Fc (LI-COR Biosciences, Lincoln, NE) in a linear range of exposure (2-5min). Relative levels of CFTR protein were quantified by densitometry of immunoblots using ImageStudioLite (LI-COR Biosciences, Lincoln, NE) (Cao et al., 2020; Erwood et al., 2020; Wu et al., 2019).

#### Mass spectrometry

Photoaffinity labeled CFTR-containing membranes were solubilized in SDS-PAGE loading buffer and proteins were separated by SDS-PAGE followed by Coumassie staining. The band corresponding to the molecular weight of CFTR was excised and the proteins were digested with trypsin according to the standard in-gel digestion procedure (Borchers et al., 2000).

Tryptic digests were desalted with ZipTips (Sigma) and analyzed by nano-LC-MS with data dependent acquisition using an Easy-nLCTM 1200 System (Thermo Scientific) online-coupled to a Q Exactive Plus (Thermo Scientific) mass spectrometer. Peptides were pre-concentrated on an AcclaimTMPepMapTM 100 C18 pre-column (3 μm particle size, 75 μm inner diameter x 2 cm length) and separated on an AcclaimTM PepMapTM 100 C18 main column (2 μm particle size, 75 μm inner diameter x 25 cm length) using a 0-50% B 50-min binary gradient (Buffer A: 0.1% FA, buffer B: 84% ACN in 0.1% FA), at a flow rate of 300 nL/min. Full MS scans were acquired from m/z 350-1,500 at a resolution of 70,000 with an automatic gain control (AGC) target value of 1x10^6^ and a maximum injection time of 120 ms. The 15 most intense precursor ions (charge states +2 - +4) were isolated with a window of m/z 1.2 and fragmented using a normalized higher-energy collisional dissociation energy of 28, and a dynamic exclusion of 40 s. The MS/MS spectra were acquired at a resolution of 17,500, using an AGC target value of 2x10^4^ and a maximum injection time of 64 ms. LC-MS data were processed using the Proteome Discoverer 2.4 software suite (Thermo Scientific) using the Sequest search engine and Percolator validation nodes against human proteome database using variable modification setting corresponding to the covalent modification with VX-770-DIAZ (2) probe (elemental addition of H_19_C_22_F_3_N_2_O_3_, mass addition of 416.1348) at any residue.

#### Preparation of human CFTR structural model

The structural model of phosphorylated, ATP-bound human CFTR (PDB: 6MSM) was used for all simulations (Zhang et al., 2018). Residues that were missing in this structure consisted of the segments connecting each MSD-NBD pair (410-434, 1174-1201), the R-domain (638-844), the extracellular loop connecting TM7 and TM8 (also known as the ECL-4; 890-899), and the C-terminal stretch after NBD2 (1452-1489). The missing segments likely were unstructured with unknown amounts of post-translational modifications. Furthermore, a number of CFTR analogs with segments removed were found to be functional, including the one without the R-domain (Bompadre et al., 2005), while the overall structure of CFTR was found to be stable over microseconds of simulation. Therefore, the missing proteogenic segments were not modeled in this study, resulting in our structural model consisting of five peptide segments. To reduce truncation artifacts in our simulations, all five peptide segments were acetylated at the N-termini and amidated at the C-termini (primary amide C-termini). Two Mg-ATP moieties from the 6MSM structure were kept at their origin positions between the NBDs. All other species present in the 6MSM structure were removed if they were not part of known regions of CFTR or Mg-ATP. To determine the appropriate starting position of the lipid bilayers, the PPM server of Orientations of Proteins in Membranes (OPM) database was used (all default settings) (Lomize et al., 2012).

#### Parametrization of ivacaftor (VX-770)

CHARMM-compatible force field parameters for VX-770 were created using the General Automated Atomic Model Parameterization (GAAMP) procedure (Huang and Roux, 2013). This technique automatically generates the parameters of atomic models of small molecules using the results from ab initio quantum mechanical (QM) calculations and is available through an automated web server (https://gaamp.lcrc.anl.gov/) and open-source repository (https://github.com/gaamp). CHARMM-compatible parameter and topology files for VX-770 were created using the webserver with -CH3 hydrogen atom equivalency and all default parameters.

#### Preparation of simulation systems containing ivacaftor: CFTR in POPC

In the first set of simulations, we intended to have ivacaftor starting in the aqueous phase. The model for CFTR with bound Mg-ATP was embedded in POPC bilayer and solvated in explicit water with 150 mM NaCl using CHARMM-GUI server (Jo et al., 2008). The hexagonal periodic unit cell configuration was adopted (starting dimensions: *a* = *b* = 11 nm, *c* = 18.5 nm, *α* = *β* = 90°, *γ* = 120°) such that 255 lipid molecules were added around the protein. No water molecules or ions were placed within the channel pore in this system. One VX-770 molecule was inserted in the aqueous phase of this system using GROMACS *insert-molecules* functionality (Mark James Abraham et al., 2015). Ten independent random insertions of VX-770 molecules were done resulting in ten configurations of POPC-embedded CFTR with a VX-770 molecule in water.

#### Preparation of simulation systems containing ivacaftor: CFTR in octanol slab

To increase the chance of sampling of VX-770 location in and out of the transmembrane region, we used octanol slab as membrane mimetic in the additional set of simulations. To prepare either type of system, a rectangular slab (periodic unit cell dimensions: *a* = *b* = 11 nm, *c* = 3.5 nm, *α* = *β* = *γ* = 90°) filled with octanol were first created using GROMACS *insert-molecules* command (Mark James Abraham et al., 2015). The box contained as many octanol molecules as practically possible to prevent the box from shrinking too much in the *xy*-directions during NPT runs. This slab/box of octanol was then subjected to energy minimization, which was then followed by 20-ns-long NVT equilibration and 20-ns-long NPT equilibration. The compressibility of the system in the *z*-direction was set to zero during the NPT step in order to maintain the thickness of the slab (Kulleperuma et al., 2013; Martin et al., 2004).

The structural model of CFTR with two bound Mg-ATP was then embedded in this equilibrated octanol slab using GROMACS tools (Mark James Abraham et al., 2015), during which octanol molecules that overlapped with protein were removed. Care was taken to make the *z*-position of the membrane mimetic to be the same as the POPC-bilayer described above. The assembled system was then placed in a bigger rectangular box (*a* & *b* are the same as equilibrated slab, *c* = 16.5 nm). The system was solvated in explicit three-point water using GROMACS *solvate* (Mark James Abraham et al., 2015). The resulting system was edited with the help of VMD to remove water molecules inside the octanol slab (Humphrey et al., 1996). Neutralizing chloride ions were added to the aqueous phase using GROMACS *genion* (Huang and Roux, 2013; Mark James Abraham et al., 2015). Finally, one VX-770 molecule was randomly inserted into the aqueous phase. Random insertion was done twenty times, resulting in twenty starting configurations for MD simulations. CHARMM parameters for octanol was available as part of the CHARMM36 force field and were obtained from CHARMM-GUI Ligand Reader (Kim et al., 2017).

#### MD Simulations

All MD simulations and analysis involving *gmx* commands were conducted using GROMACS 2018.3 (Mark James Abraham et al., 2015). The CHARMM36 force field for protein, lipids, ions, ATP and the TIP3P water model were used (Huang and MacKerell, 2013; Klauda et al., 2010). All simulations ran in the *NpT* ensemble (*T* = 300 K, *p* = 1 atm) at 2 fs integration timesteps. Constant temperature was maintained using Nosé–Hoover thermostat (τ_T_ = 0.5) (Hoover, 1985); constant pressure was maintained using Parrinello-Rahman barostat (τ_p_ = 2.0). Semi-isotropic pressure coupling scheme was used for all simulations. For POPC-embedded systems, isothermal compressibility was set to 4.5×10^-5^ bar^-1^ in both *xy*-direction and *z*-direction. For octanol-embedded systems, isothermal compressibility in the *xy*-direction was also 4.5×10^-5^ bar^-1^ while being set to zero in the *z*-direction. This setting had been adopted in a number of cases in other studies that used octane slab as the membrane mimetic (Kulleperuma et al., 2013, Martin et al., 2004). We found this setting to be necessary to maintain the integrity of octanol slab. Nonbonded interactions were calculated using Verlet neighbor lists (Verlet, 1967). Lennard-Jones interactions were cut off at 1.2 nm and a force-based switching function with a range of 1.0 nm was used. Particle-mesh Ewald (PME) method was used to compute electrostatic interactions with a real-space cut-off of 1.2 nm (Essmann et al., 1995). LINCS was used to constrain covalent bonds involving hydrogen (Hess, 2008).

After insertion of VX-770, all systems were subjected to steepest descent energy minimization until maximum force was below 1000 kJ/mol/nm, followed by *NpT*-equilibration. Random velocities were generated at the beginning of the *NpT*-equilibration phase. For systems with POPC-embedded CFTR, *NpT*-equilibration was done in three 10 ns-stages, successively with protein heavy atoms, protein backbone atoms, and protein Cα atoms restrained. For systems with octanol-embedded CFTR, *NpT*-equilibration was done once with protein backbone atoms restrained for 50 ns. Additionally, isothermal compressibility in the *z*-direction was set to zero for this system. System configurations at the end of *NpT*-equilibration phase were subjected to production runs in the *NpT* ensemble. New random velocities were generated at the beginning of production runs. For systems with POPC, each production run was 1-μs-long. For systems with octanol, each production run was 500-ns-long. In total, 30 MD trajectories were obtained, from which 10-μs of aggregate simulation time was obtained for either of POPC-embedded and octanol-embedded systems.

#### Analysis of MD simulations

Analysis was performed on all simulation frames spaced by 1 ns from each simulation repeat. RMSD map for VX-770 were computed using homemade Python scripts aided by the MDTraj package (McGibbon et al., 2015). All rendering of images was done using VMD (Humphrey et al., 1996).

To identify binding events, we searched within our simulation trajectories for time periods where VX-770 remained relatively stationary: not undergoing significant changes in position, pose, and conformation. To do this, for each trajectory, after aligning the conformations of CFTR according to the Cα atoms of the CFTR structure in the first frame were aligned. For each frame in a given simulation trajectory of *N* frames, the root-mean-squared-distances (RMSD) of non-hydrogen atoms of VX-770 between this frame and all *N*-1 frames are computed. This results in an *N*×*N* square matrix of RMSD values indicating time intervals when putative binding events occurred (Grossfield et al., 2018). The square matrix was plotted as an RMSD map such that values less than 5 Å are color-indicated. A non-white squared cluster indicates low RMSD values – a time period when few movements and conformational changes of VX-770 occur, suggesting a potential binding event. VMD was used to visualize and aid grouping of binding events, as well as excluding low RMSD clusters that are either not actually binding to the protein or having single occurrences with short binding durations. Potential energies of VX-770 binding were calculated using GROMACS *energy* functionality (Mark James Abraham et al., 2015).

#### CFTR channel function studies using the membrane potential sensitive dye: FLIPR

HEK cells seeded in 96-well plates and transiently transfected with WT-, K1041A, F1052A, Y304A, F312A-CFTR constructs. After 48h of transfection, the cells were washed with phosphate buffered saline and preincubated for 35 min at 37 °C with blue membrane potential dye (dissolved in chloride-free buffer as described) (Laselva et al., 2020d; Laselva et al., 2020e). The plate was read in a fluorescence plate reader (SpectraMaxi3; Molecular Devices) at 37 °C (excitation: 530 nm, emission: 560 nm). Following the first 5 min of the baseline recording, CFTR was stimulated using the cAMP agonist forskolin (1μM, Sigma–Aldrich) +/- 1μM VX-770 or 1μM VX-770-Biot for 10 min. To further determine that measurements were CFTR-specific, the CFTR inhibitor CFTRinh-172 (10 μM, Cystic Fibrosis Foundation Therapeutics) was added to deactivate CFTR. CFTR-mediated depolarization of the plasma membrane was detected as an increase in fluorescence and hyperpolarization (or repolarization) as a decrease (Laselva et al., 2020a).
