# Supplementary material for: Identification of binding sites for ivacaftor on the cystic fibrosis transmembrane conductance regulator
Source: iScience. 2021 May 15;24(6):102542. doi: 10.1016/j.isci.2021.102542 (PMC8184517; doi:10.1016/j.isci.2021.102542)
Supplement: Document S1. Figures S1–S4 [file mmc1.pdf]

iScience, Volume *24*

Supplemental information

Identification of binding sites for ivacaftor on the cystic fibrosis trans-membrane conductance regulator

Onofrio Laselva, Zafar Qureshi, Zhi-Wei Zeng, Evgeniy V. Petrotchenko, Mohabir Ramjeesingh, C. Michael Hamilton, Ling-Jun Huan, Christoph H. Borchers, Régis Pomès, Robert Young, and Christine E. Bear

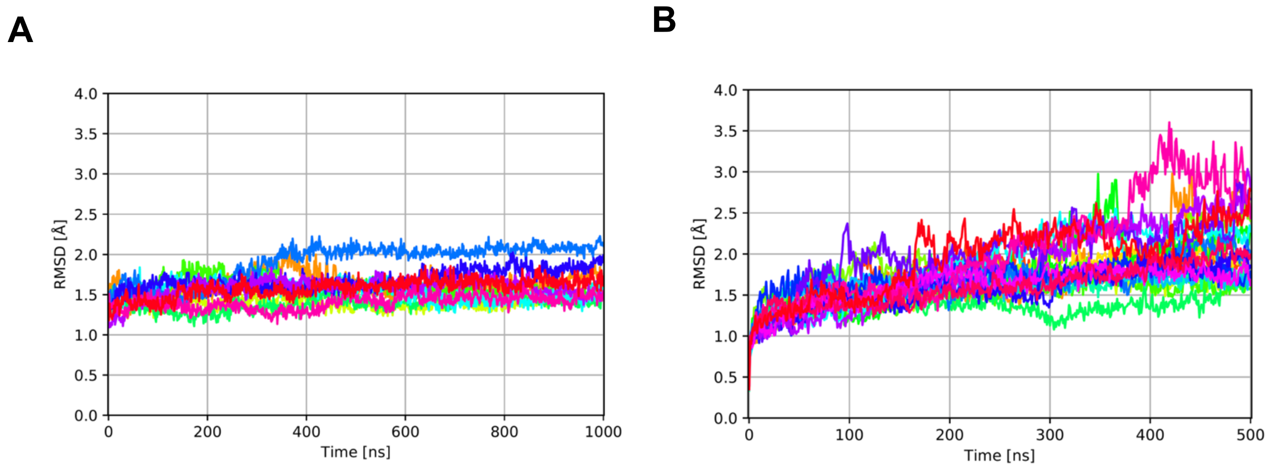

FIGURE S1: Root-mean-squared deviations (RMSDs) of MSDs of CFTR in trajectories from the ATP-bound, NBD-dimerized human CFTR structure (PDB: 6MSM). Related to Figure 4. **(A)** POPC-embedded MD simulations; and (**B**) octanol-embedded MD simulations.

A

System	Traj. Number	Time Interval (ns)	Location	Location Type	Total Non-covalent (kJ/mol)
POPC	3	656-820	a	NBD2	-207.0±22.0
OCTANOL	15	91-349	b	NBD1-NBD2	-186.6±28.6
POPC	4	129-725	b	NBD1-NBD2	-183.8±29.8
POPC	5	447-574	c	MSD2-Lasso	-181.8±35.1
OCTANOL	15	349-412	b	NBD1-NBD2	-160.2±31.2
POPC	3	831-1000	a	NBD2	-157.1±19.9
POPC	5	857-1000	c	MSD2-Lasso	-154.2±16.1
POPC	9	127-230	d	MSD-NBD1	-151.4±27.3
OCTANOL	7	0-500	e	NBD1	-135.1±35.5
POPC	10	390-1000	c	MSD2-Lasso	-132.9±35.1
OCTANOL	18	20-180	c	MSD2-Lasso	-129.8±49.4
POPC	4	800-1000	b	NBD1-NBD2	-127.4±16.7
OCTANOL	6	0-460	c	MSD2-Lasso	-119.3±25.8
POPC	10	150-348	e	MSD-NBD1	-118.3±16.6
POPC	6	575-1000	b	NBD1-NBD2	-117.4±23.2
POPC	8	216-1000	c	MSD2-Lasso	-117.0±18.4
OCTANOL	18	348-500	c	MSD2-Lasso	-116.6±26.2
POPC	6	444-573	f	NBD2	-113.1±16.3
OCTANOL	18	182-345	c	MSD2-Lasso	-111.9±16.8
POPC	1	803-1000	f	NBD2	-107.5±13.2
POPC	2	86-980	g	MSD1	-107.1±36.4
POPC	9	278-1000	d	MSD-NBD1	-96.0±14.7

B

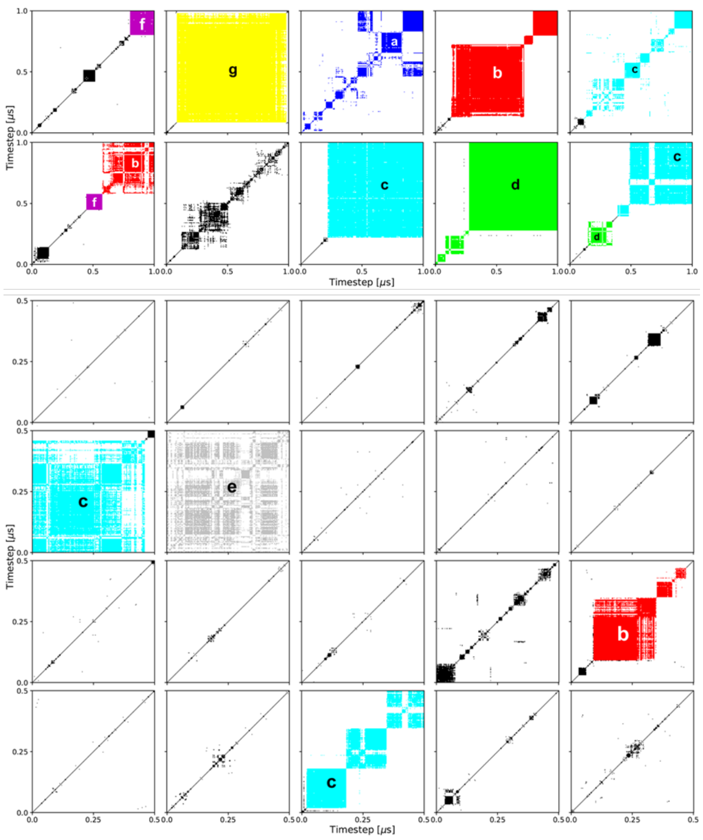

FIGURE S2: **Molecular dynamics simulations. Related to Figure 4.**
(**A**) Ranking of binding events of VX-770. The table is sorted based on total non-covalent potential energy of interaction between VX-770 and CFTR (coulombic+ Lennard Jones). Energy values are displayed as mean (over the time interval) ± 1x standard deviation. (**B**) Binding events in POPC-embedded and OCTANOL-embedded systems visualized for all simulation trajectories on RMSD maps. Binding of VX-770 are colour-coded according to the table in A) and labelled with lowercase letters denoting their locations of binding. Regions coloured in black are not considered binding events based on visual inspection of trajectories.

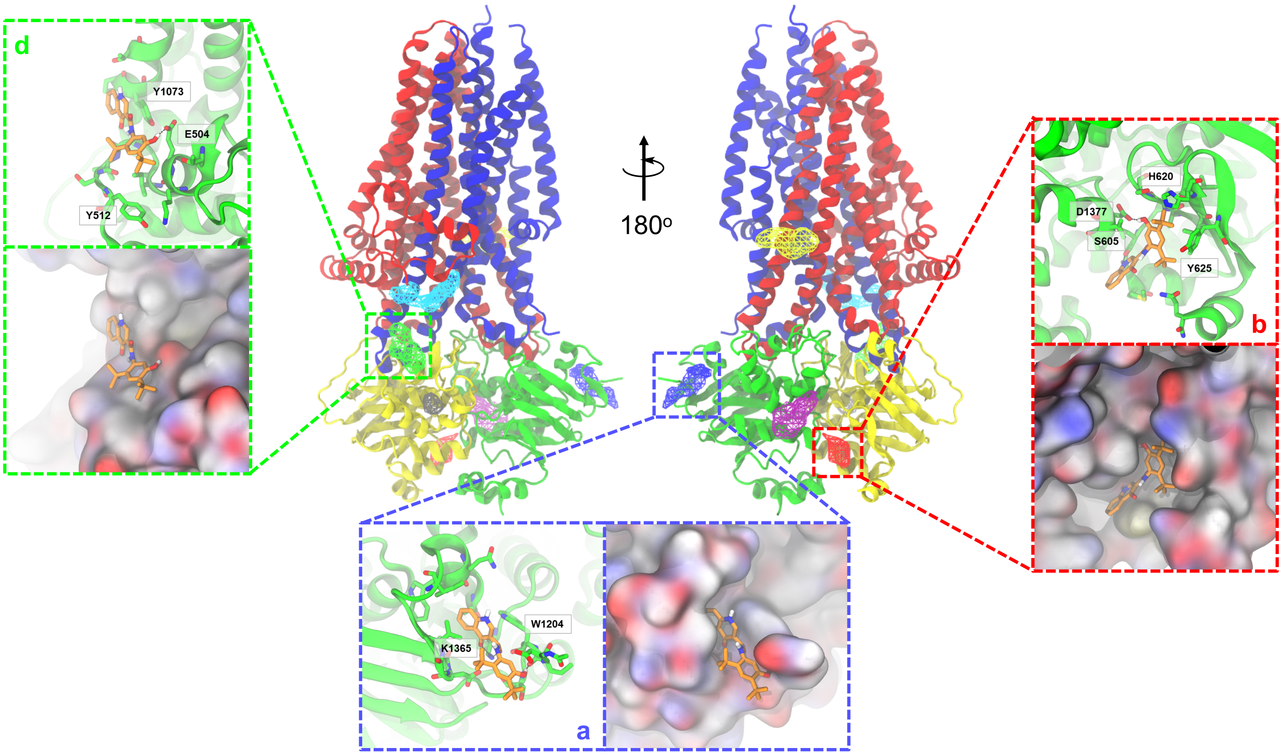

FIGURE S3: **Snapshots of VX-770 binding in at locations a, b, and d, as shown in Table S2-A**. **Related to Figure 4.**

The locations on CFTR are colour-coded. Four structured domains are color coded (red: Lasso motif-MSD1; yellow: NBD1; blue: MSD2; green: NBD2). Representative snapshots were shown in both cartoon and surface views.

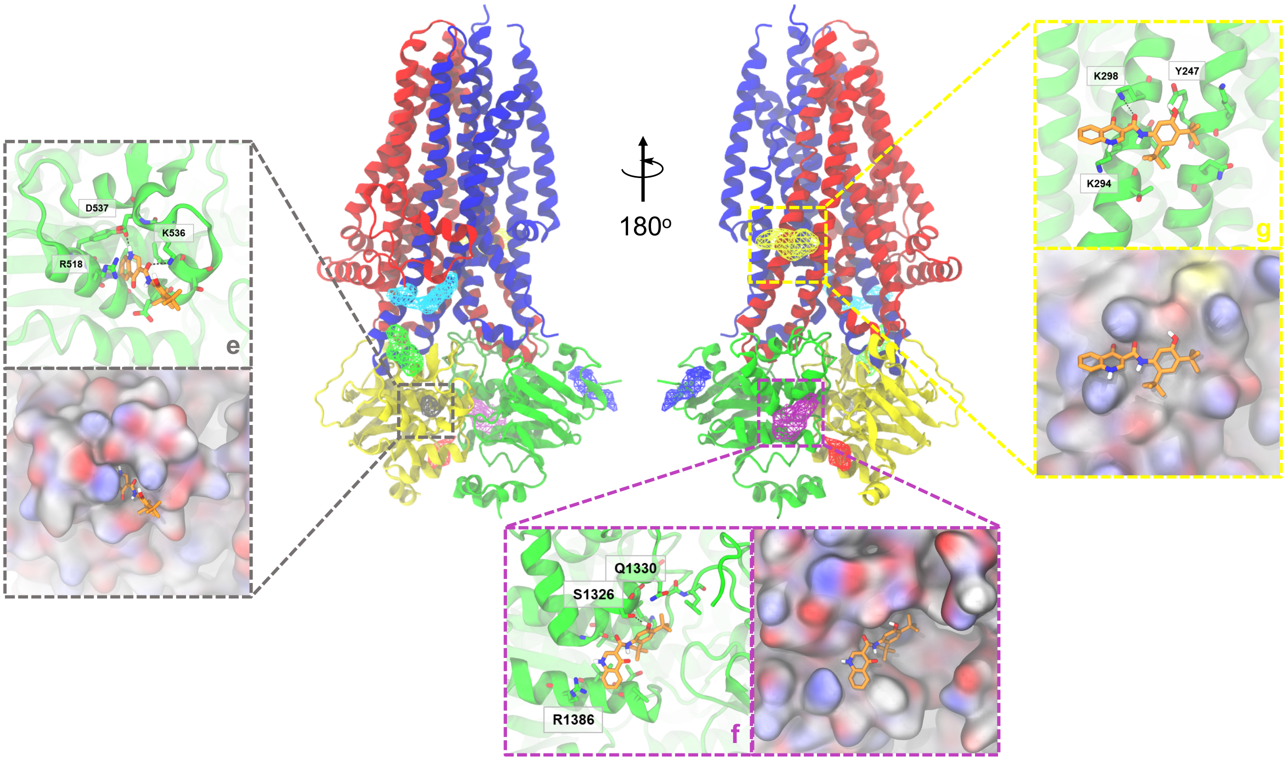

FIGURE S4: Snapshots of VX-770 binding in at locations e, f, and g, as shown in Table S2-A. Related to Figure 4.

The locations on CFTR are colour-coded. Four structured domains are color coded (red: Lasso motif-MSD1; yellow: NBD1; blue: MSD2; green: NBD2). Representative snapshots were shown in both cartoon and surface views.

Supplementary Chemistry Methods:

Two photoaffinity labeling probes were used in this study based on the structure of ivacaftor (VX-770, **1**, ***Figure 1***). The synthesis and characterization of the photoaffinity labeling probe VX-770-DIAZ (**2**) was reported recently,[3] and was used in this work for in the labeling and mass spectrometry studies with wt-CFTR. Photoaffinity labeling probe VX-770-BIOT (**3**) was prepared in a similar manner as **2**, featuring a diazirine photo-reactive group, and biotin as a reporter tag. VX-770-BIOT was used for labeling wt-CFTR and CFTR domain constructs for use in pull-down assays.

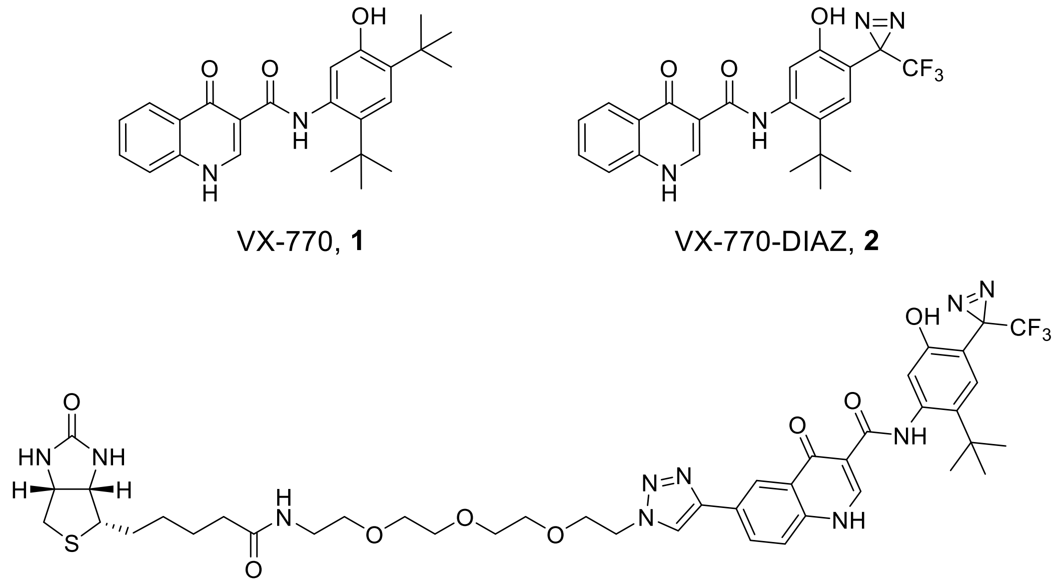

VX-770-BIOT, **3**

Figure 1. Structures of ivacaftor (VX-770, **1**) and photoaffinity labeling probes VX-770-DIAZ (**2**) featuring a diazirine, and VX-770-BIOT (**3**) incorporating a biotin reporter tag

The synthesis of **3** (***Scheme 1***) began with a HATU-mediated coupling of acid **4** and aniline **5** to give amide **6**. In a Suzuki reaction using $PdCl_2(PPh_3)_2$ and ethynyltrimethylsilane, the iodo group of **6** was converted to a TMS-protected acetylene group (**7**). After first reacting **7** with two equivalents of MeLi, a lithium-halogen exchange of the bromine atom was performed by treatment with *n*-BuLi, and the resulting tri-anion was reacted with ethyl trifluoroacetate affording the trifluoromethylketone (**8**) as a mixture of the ketone, hydrate and related hemi-ketal species. Reaction of the crude mixture of **8** with hydroxylamine hydrochloride in pyridine/methanol gave oxime **9**, which was isolated by trituration in DCM/hexanes to give a 2:1 mixture of (unassigned) E/Z isomers. Tosylation of oxime **9** with *p*-Tosyl chloride, and subsequent reaction with liquid ammonia effected the transformation to the diaziridine, followed by deprotection of the TMS-acetylene with K_2CO_3 in methanol, to provide diaziridine **10**. A Cu mediated "click" reaction of **10** with Biotin-PEG3-azide and TPTA gave the oxidized diazirine **11** as the major species. The final deprotection of the phenol group of **10** with methanolic HCl gave clean

conversion to VX-770-BIOT (**3**) without further purification. Solutions of **3** in DMSO were stored at -80 °C.

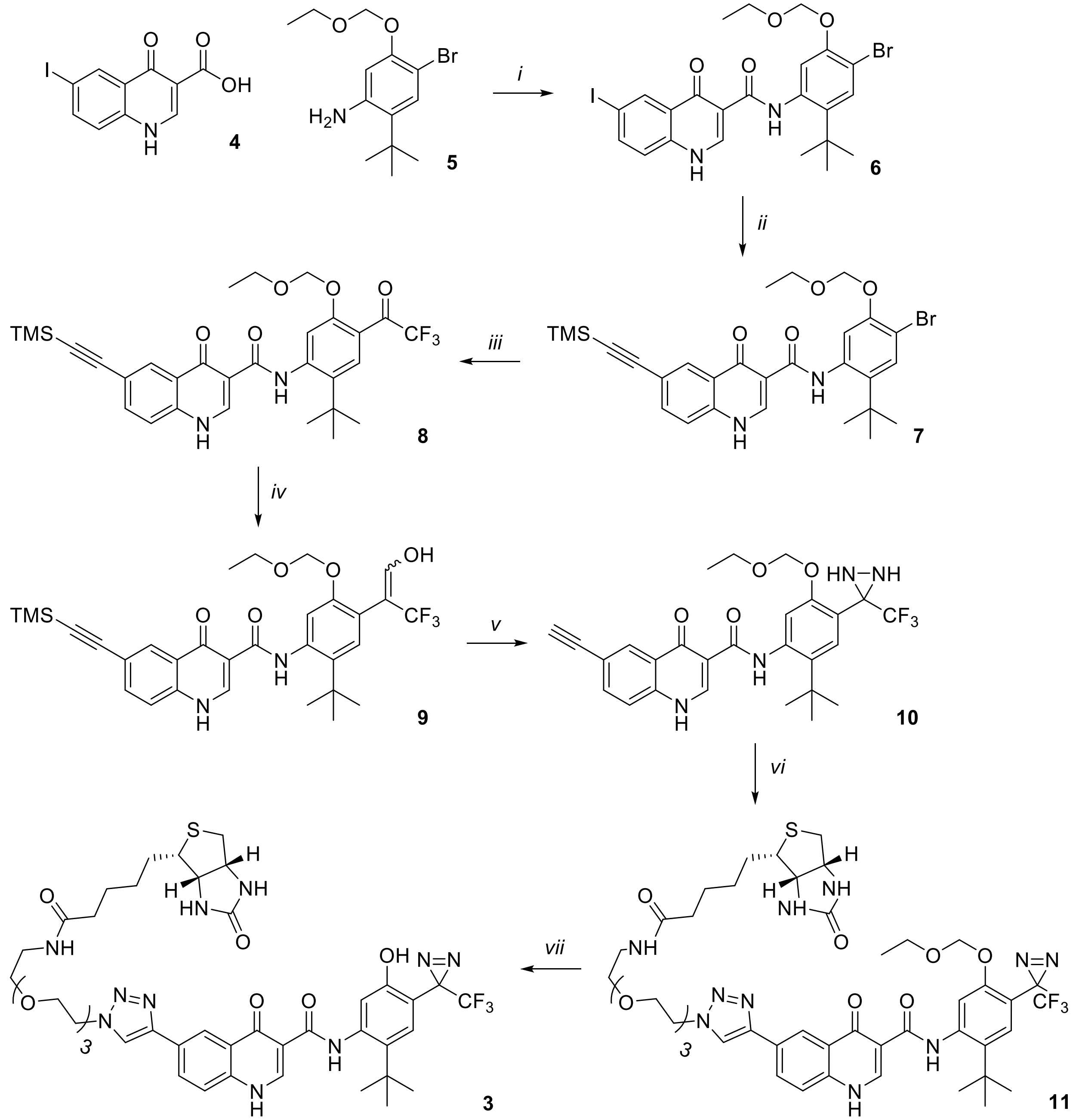

Scheme 1. Synthesis of VX-770-BIOT (**3**) [a]Reagents and conditions: (*i*) HATU, DMAP, DIPEA, HOBt, DMF, 60 °C, overnight; (*ii*) ethynyltrimethylsilane, $PdCl_2(PPh_3)_2$, CuI, DIPEA, DMF, rt. 1h; (*iii*) a)MeLi, THF, -40 °C, 30 min; b) *n*-BuLi, -78 °C, 1h; c) Ethyl trifluoroacetate, -78 °C to rt.; (*iv*) NH2OH.HCl, 2:1 Pyridine/MeOH, 80 °C, overnight; (*v*) a) TsCl, DIPEA, DCM, 0 °C, 1h; b) NH_3 (*l*), DCM, -78°C to rt.; (*vi*) Biotin-PEG3-azide, CuI, TBTA, Et_3N, DMF, 60 °C, 1h; (*vii*) Methanolic HCl, rt., 1h.

Experimental

General Chemistry

THF was distilled from Na and benzophenone under N_2. All other reagents and solvents were used as received from commercial suppliers unless otherwise stated. Melting points were measured on a Fisher-Johns melting point apparatus and are uncorrected. Microwave reactions were conducted in a Biotage Initiator®. Flash chromatography was performed with a Biotage Isolera One® system using SiliCycle SiliaSep™ Cartridges of indicated size and solvent gradient. ^{1}H, ^{19}F and ^{13}C NMR spectra were recorded with Bruker Avance II™600 MHz, Bruker Avance III™500 MHz, or Bruker Avance III™400 MHz. ^{1}H and ^{13}C NMR spectra were referenced to the indicated residual solvent signals[1] and processing of the spectra was performed with MestRecNova™software (Note: numbering of atoms for the purposes of signal assignment did not follow IUPAC numbering schemes). The high-resolution mass spectra were recorded with an ESI ion source on an Agilent™Time-of-Flight LC/MS mass spectrometer (Model 6210) using a HaloC18(2.150 mm) water-ACN (5 mM NH4OAc) gradient. All HPLC analyses were performed utilizing an Advanced Materials Technologies Halo™ C18 reverse-phase analytical column (4.6 x 50 mm, 5 µm) with a water-ACN (5 mM NH4OAc) gradient. All preparative HPLC purifications were carried out using a Phenomenex Kinetex C18 reverse-phase preparatory column (21.2 x 150 mm, 5 µm) with a water-ACN (5 mM NH4OAc) gradient. ATR spectra were collected with a Perkin Elmer Spectrum Two™FT-IR spectrometer, and absorption bands were designated as weak (w), broad (br), medium (m), or strong (s). Photo-decomposition studies were performed with a UVP Mineralight©lamp (115 V, 60 Hz, 0.16 A) and irradiated at 365 nm (long UV wavelength setting).

N-(4-bromo-2-(*tert*-butyl)-5-(ethoxymethoxy)phenyl)-6-iodo-4-oxo-1,4-dihydroquinoline-3-carboxamide (**6**)

6-iodo-4-oxo-1,4-dihydroquinoline-3-carboxylic acid (prepared according to Chen et al.[2]) (3.47 g, 11 mmol), 4-bromo-2-(*tert*-butyl)-5-(ethoxymethoxy)aniline (prepared according to Hamilton et al.[3]) (5.00 g, 16.5 mmol), HATU (8.36g, 22 mmol, 2 equiv), HOBt (1.49g, 11mmol, 1 equiv), DMAP (134 mg, 1.1 mmol, 0.1 equiv), DIPEA (5.7 ml, 33 mmol, 3 equiv) were dissolved in DMF (50 ml) and heated to 60 ^{o}C overnight. The solvent was removed and the crude black oil was dissolved in ethyl acetate, washed with saturated $NaHCO_3$, H_2O, and brine then dried with $MgSO_4$, filtered and concentrated. The crude product was dissolved in $CHCl_3$/MeOH then product precipitated with Hexanes to give **6** (5.27 g, 80 % yield) as a pale red solid. ^{1}H NMR (400 MHz, Acetone-d_6): δ 12.04 (s, 1H), 9.00 (s, 1H), 8.73 (d, J = 2.0 Hz, 1H), 8.04 (dd, J = 8.7, 2.1 Hz, 1H), 7.81 (s, 1H), 7.60 (s, 1H), 7.46 (d, J = 8.7 Hz, 1H), 5.28 (s, 2H), 3.73 (q, J = 7.1 Hz, 2H), 1.50 (s, 9H), 1.16 (t, J = 7.1 Hz, 3H). ^{13}C NMR (101 MHz, Acetone-d_6): δ 175.53, 162.85, 151.77, 144.82, 141.22, 138.70, 137.73, 136.26, 134.80, 130.51, 128.24, 121.06, 115.69, 112.01, 107.98, 93.82, 88.90, 64.29, 33.99, 29.81, 14.53. HRMS: *m/z* calculated for $C_{23}H_{24}{}^{79}BrIN_2O_4$: 596.9891 $(M-H)^-$; Found 596.9886.

N-(4-bromo-2-(*tert*-butyl)-5-(ethoxymethoxy)phenyl)-4-oxo-6-((trimethylsilyl)ethynyl)-1,4-dihydroquinoline-3-carboxamide (**7**)

A mixture of **6** (1.2 g, 2 mmol), ethynyltrimethylsilane (0.41 ml, 3 mmol, 1.5 equiv), $PdCl_2(PPh_3)_2$ (140 mg, 0.2 mmol, 0.1 equiv), CuI (114 mg, 0.6 mmol, 0.3 equiv) was dissolved in DIPEA/DMF (20 ml, 1:1) and stirred at room temperature for 1h. The solvent was removed and the crude solid was triturated in MeOH/H_2O to give **7** (1.05 g, 95% yield) as a brown solid. ^{1}H NMR (400 MHz, Acetone-d_6): δ 12.01 (s, 1H), 11.94 (s, 1H), 9.02 – 8.92 (m, 1H), 8.49 (dd, *J* = 1.9, 0.6 Hz, 1H), 7.90 (s, 1H), 7.83 (dd, *J* = 8.6, 1.9 Hz, 1H), 7.78 (dd, *J* = 8.6, 0.7 Hz, 1H), 7.58 (s, 1H), 5.31 (s, 2H), 3.78 (q, *J* = 7.1 Hz, 2H), 1.51 (s, 9H), 1.20 (t, *J* = 7.1 Hz, 3H), 0.29 (s, 9H). ^{13}C NMR (101 MHz, Acetone-d_6): δ 176.22, 162.64, 151.73, 144.70, 139.09, 137.44, 136.38, 135.32, 130.43, 129.59, 126.43, 119.86, 119.45, 115.43, 112.14, 107.70, 103.84, 94.93, 93.84, 64.27, 33.96, 29.78, 14.53, -0.98. HRMS: *m/z* calculated for $C_{28}H_{33}{}^{79}BrN_2O_4Si$: 567.1320 (M-H); Found 567.1337.

N-(2-(*tert*-butyl)-5-(ethoxymethoxy)-4-(2,2,2-trifluoroacetyl)phenyl)-4-oxo-6-((trimethylsilyl)ethynyl)-1,4-dihydroquinoline-3-carboxamide (**8**)

A solution of **7** (800mg, 1.4mmol) dissolved in dry THF (8ml) under N_2 was cooled to -40 °C. MeLi (2.0ml, 3.2mmol, 2.3eq, 1.6M in Et_2O) was added dropwise changing the yellow solution to red. CH_4 (*g*) evolution was monitored with a bubbler. When gas evolution ceased (10 – 20 min) *n*-BuLi (1.08 ml, 2.4 mmol, 1.2 equiv, 1.58 M in Hexanes) was added causing a precipitate to form. The viscous slurry was stirred for 10 min then ethyl trifluoroacetate (4 ml, 34 mmol, 24 equiv) was added and the reaction was warmed to room temperature. After stirring 5 min at room temperature the reaction was quenched with aqueous NH_4Cl, extracted with ethyl acetate, dried with $MgSO_4$, filtered and concentrated to give **8** (591 mg, crude) as a mixture of ketone, hemi-ketal, ketal, along with debrominated side product and starting material. The mixture was used in the following reaction without purification. HRMS: *m/z* calculated for $C_{30}H_{34}F_3N_3O_5Si$: 585.2038(M-H); Found 585.2063

N-(2-(*tert*-butyl)-5-(ethoxymethoxy)-4-(2,2,2-trifluoro-1-(hydroxyimino)ethyl)phenyl)-4-oxo-6-((trimethylsilyl)ethynyl)-1,4-dihydroquinoline-3-carboxamide (**9**)

The crude mixture of trifluoromethylketone **8** (591mg) and $NH_2OH{\cdot}HCl$ (591 mg, 8.4 mmol) was dissolved in pyridine (20 ml) and heated to 80 °C overnight. The reaction was concentrated and the crude material was triturated in MeOH/H_2O to give oxime **9** (563 mg) which was used in the following reaction without further purification. HRMS: *m/z* calculated for $C_{30}H_{34}F_3N_3O_5Si$: 600.2147(M-H); Found 600.2136

N-(2-(*tert*-butyl)-5-(ethoxymethoxy)-4-(3-(trifluoromethyl)diaziridin-3-yl)phenyl)-6-ethynyl-4-oxo-1,4-dihydroquinoline-3-carboxamide (**10**)

Oxime **9** (563 mg) was dissolved in CH_2Cl_2 (10 ml). *p*-TsCl (357 mg, 1.87 mmol) was added followed by DIPEA (0.49 ml, 2.8 mmol) and the reaction was stirred at room temperature for 1h. The reaction was then cooled to -78 °C and NH_3 (*l*) (100ml) was condensed directly into the reaction mixture. The reaction was allowed to warm to room temperature overnight allowing the NH_3 (*l*) to evaporate. The reaction was diluted with CH_2Cl_2, washed with aqueous NH_4Cl, dried with $MgSO_4$, filtered and concentrated. The crude product was dissolved in MeOH (20ml) and treated with K_2CO_3 (1eq w/w) until complete deprotection of the silyl group. The excess K_2CO_3 was filtered off and the filtrate concentrated. The

crude material was purified by column chromatography (50→100% EA/Hex) to give **10** (196 mg, 0.37 mmol, 26 % over 5 steps) as a white amorphous solid. ^{1}H NMR (400 MHz, Acetone-*d$_6$*): δ 12.03 (s, 1H), 8.97 (s, 1H), 8.53 (d, J = 1.6 Hz, 1H), 7.96 – 7.79 (m, 3H), 7.57 (s, 1H), 5.41 – 5.19 (m, 2H), 3.83 (s, 1H), 3.78 (q, J = 7.1 Hz, 2H), 3.46 – 3.30 (m, 2H), 1.51 (s, 9H), 1.21 (t, J = 7.1 Hz, 3H). ^{19}F NMR (376 MHz, Acetone-*d$_6$*): δ -76.95. ^{13}C NMR (101 MHz, Acetone-*d$_6$*): δ 176.22, 162.72, 154.68, 144.73, 139.26, 135.55, 134.90, 129.71, 128.81, 126.51, 119.48, 119.10, 113.30, 112.28, 93.34, 82.28, 79.14, 64.06, 33.89, 29.91, 14.48.**Not all peaks observed due to poor signal to noise ratio. HRMS: *m/z* calculated for $C_{27}H_{27}F_3N_4O_4$: 527.1912(M-H); Found 527.1902.

N-(2-(tert-butyl)-5-(ethoxymethoxy)-4-(3-(trifluoromethyl)-3H-diazirin-3-yl)phenyl)-4-oxo-6-(1-(13-oxo-17-((3aS,4S,6aR)-2-oxohexahydro-1H-thieno[3,4-d]imidazol-4-yl)-3,6,9-trioxa-12-azaheptadecyl)-1H-1,2,3-triazol-4-yl)-1,4-dihydroquinoline-3-carboxamide (**11**)

Diaziridine **10** (5.3mg, 10umol), Biotin-PEG3-azide (4.4mg, 10 µmol, 1eq), CuI (1.9mg, 10 µmol, 1eq), TBTA (5.3mg, 10 µmol, 1eq) and Et_3N (7ul, 50 µmol, 5eq) were dissolved in DMF (1ml, degassed) and heated to 60 °C for 1h. The reaction was cooled to RT and the mixture was directly purified by reverse phase HPLC (60→100% MeOH/H_2O, 1% Formic acid over 20mins) on a Kinetex 5µm, C18 column, 150 X 21.1mm to give N-(2-(tert-butyl)-5-(ethoxymethoxy)-4-(3-(trifluoromethyl)-3H-diazirin-3-yl)phenyl)-4-oxo-6-(1-(13-oxo-17-((3aS,4S,6aR)-2-oxohexahydro-1H-thieno[3,4-d]imidazol-4-yl)-3,6,9-trioxa-12-azaheptadecyl)-1H-1,2,3-triazol-4-yl)-1,4-dihydroquinoline-3-carboxamide (**11**) (3mg, 31 %) as a white solid. ^{1}H NMR (600 MHz, Acetone-*d*$_6$): δ 12.63 (s, 1H), 12.26 (s, 1H), 8.93 (s, 1H), 8.90 (d, *J* = 1.7 Hz, 1H), 8.64 (s, 1H), 8.35 (d, *J* = 8.6 Hz, 1H), 8.05 (s, 1H), 7.90 (d, *J* = 8.6 Hz, 1H), 7.54 (s, 1H), 7.06 (s, 1H), 6.25 (s, 1H), 5.91 (s, 1H), 5.40 (s, 2H), 4.68 (t, *J* = 5.0 Hz, 2H), 4.55 (t, *J* = 6.1 Hz, 1H), 4.35 (t, *J* = 6.2 Hz, 1H), 4.00 (t, *J* = 5.1 Hz, 2H), 3.81 (q, *J* = 7.1 Hz, 2H), 3.67 – 3.63 (m, 2H), 3.61 – 3.58 (m, 2H), 3.54 – 3.57 (m, 2H), 3.53 – 3.50 (m, 2H), 3.36 – 3.43 (m, 2H), 3.28 – 3.14 (m, 3H), 2.94 (dd, *J* = 12.5, 5.1 Hz, 1H), 2.71 – 2.80 (m, 4H), 2.08 – 2.12 (m, 2H), 1.74 (dq, *J* = 12.7, 6.4 Hz, 1H), 1.54 (s, 9H), 1.34 – 1.46 (m, 2H), 1.22 (t, *J* = 7.1 Hz, 3H). ^{19}F NMR (376 MHz, Acetone-*d*$_6$): δ -69.71. ^{13}C NMR (151 MHz, Acetone-*d*$_6$): δ 176.9, 172.3, 163.3, 163.1, 155.6, 145.9, 144.4, 140.1, 139.0, 135.5, 130.1, 128.7, 128.0, 127.0, 122.4 (q, *J* = 274.3 Hz), 122.1, 121.9, 120.00, 113.2, 113.0, 111.6, 111.4, 93.1, 70.2, 70.2, 70.2, 70.0, 69.5, 69.0, 64.3, 61.5, 60.1, 55.5, 50.1, 40.1, 38.9, 38.7, 34.8, 34.1, 29.8, 27.8, 27.7, 26.48 (q, *J* = 42.2 Hz), 25.4, 14.6. HRMS: *m/z* calculated for $C_{45}H_{57}F_3N_{10}O_9S$: 971.4056 $(M+H)^+$; Found 971.4036.

N-(2-(tert-butyl)-5-hydroxy-4-(3-(trifluoromethyl)-3H-diazirin-3-yl)phenyl)-4-oxo-6-(1-(13-oxo-17-((3aS,4S,6aR)-2-oxohexahydro-1H-thieno[3,4-d]imidazol-4-yl)-3,6,9-trioxa-12-azaheptadecyl)-1H-1,2,3-triazol-4-yl)-1,4-dihydroquinoline-3-carboxamide (**3**)

The protected compound **11** was treated with sat. HCl in MeOH at room temperature. The progress of the reaction was monitored by HPLC, then the reaction was concentrated to give the **3** which was stored as a DMSO solution at -80 °C in the dark. Quick decomposition was observed when attempting full characterization. ^{19}F NMR (471 MHz, $CDCl_3$) δ -66.6. HRMS: *m/z* calculated for $C_{42}H_{51}F_3N_{10}O_8S$: 913.3637 $(M+H)^+$; Found 913.3596.

References:

(1) Gottlieb, H. E.; Kotlyar, V.; Nudelman, A. NMR Chemical Shifts of Common Laboratory Solvents as Trace Impurities. *J. Org. Chem.* **1997**, *62* (21), 7512–7515. https://doi.org/10.1021/jo971176v.

(2) Chen, Y. L.; Zacharias, J.; Vince, R.; Geraghty, R. J.; Wang, Z. C-6 Aryl Substituted 4-Quinolone-3-Carboxylic Acids as Inhibitors of Hepatitis C Virus. *Bioorganic Med. Chem.* **2012**, *20* (15), 4790–4800. https://doi.org/10.1016/j.bmc.2012.05.066.

(3) Hamilton, C. M.; Hung, M.; Chen, G.; Qureshi, Z.; Thompson, J. R.; Sun, B.; Bear, C. E.; Young, R. N. Synthesis and Characterization of a Photoaffinity Labelling Probe Based on the Structure of the Cystic Fibrosis Drug Ivacaftor. *Tetrahedron* **2018**, *74* (38), 5528–5538. https://doi.org/10.1016/j.tet.2018.06.016.

Supplemental Information Data File

This file contains the supplementary information for the characterization of compounds, as well as the MS data for the HSA photolabeling and competition experiments using **2**, and the MS/MS data used to identify labeled HSA peptide fragments.

Sections

1 Compound characterization data 8

1.1 *N-(4-bromo-2-(tert-butyl)-5-(ethoxymethoxy)phenyl)-6-iodo-4-oxo-1,4-dihydroquinoline-3-carboxamide (**6**)* 8

1.2 *N-(4-bromo-2-(tert-butyl)-5-(ethoxymethoxy)phenyl)-4-oxo-6-((trimethylsilyl)ethynyl)-1,4-dihydroquinoline-3-carboxamide (7)* 10

1.3 *N-(2-(tert-butyl)-5-(ethoxymethoxy)-4-(2,2,2-trifluoroacetyl)phenyl)-4-oxo-6-((trimethylsilyl)ethynyl)-1,4-dihydroquinoline-3-carboxamide* (**8**) 12

1.4 *NN-(2-(tert-butyl)-5-(ethoxymethoxy)-4-(2,2,2-trifluoro-1-(hydroxyimino)ethyl)phenyl)-4-oxo-6-((trimethylsilyl)ethynyl)-1,4-dihydroquinoline-3-carboxamide (9)* 13

1.5 *N-(2-(tert-butyl)-5-(ethoxymethoxy)-4-(3-(trifluoromethyl)diaziridin-3-yl)phenyl)-6-ethynyl-4-oxo-1,4-dihydroquinoline-3-carboxamide (**10**)* 15

1.6 *N-(2-(tert-butyl)-5-(ethoxymethoxy)-4-(3-(trifluoromethyl)-3H-diazirin-3-yl)phenyl)-4-oxo-6-(1-(13-oxo-17-((3aS,4S,6aR)-2-oxohexahydro-1H-thieno[3,4-d]imidazol-4-yl)-3,6,9-trioxa-12-azaheptadecyl)-1H-1,2,3-triazol-4-yl)-1,4-dihydroquinoline-3-carboxamide* (**11**) 18

1.7 *N-(2-(tert-butyl)-5-(ethoxymethoxy)-4-(3-(trifluoromethyl)-3H-diazirin-3-yl)phenyl)-4-oxo-6-(1-(13-oxo-17-((3aS,4S,6aR)-2-oxohexahydro-1H-thieno[3,4-d]imidazol-4-yl)-3,6,9-trioxa-12-azaheptadecyl)-1H-1,2,3-triazol-4-yl)-1,4-dihydroquinoline-3-carboxamide* (VX-770-BIOT, **3**) 21

2 Labeling of BSA with VX-770-BIOT (3) 24

1. Compound characterization data

This section contains the relevant characterization data for the series of novel compounds that were generated during the synthesis of compound **2**.

1.1. *N-(4-bromo-2-(tert-butyl)-5-(ethoxymethoxy)phenyl)-6-iodo-4-oxo-1,4-dihydroquinoline-3-carboxamide (**6**)*

Chemical Formula: $C_{23}H_{24}BrIN_2O_4$
Molecular Weight: 599.2635
Spectra provided: ^1H NMR , ^{13}C NMR

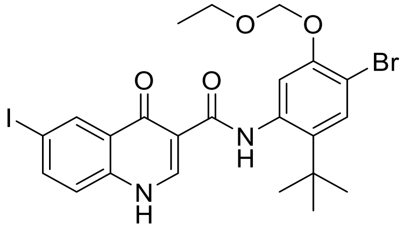

^1H NMR (Acetone-D_6):

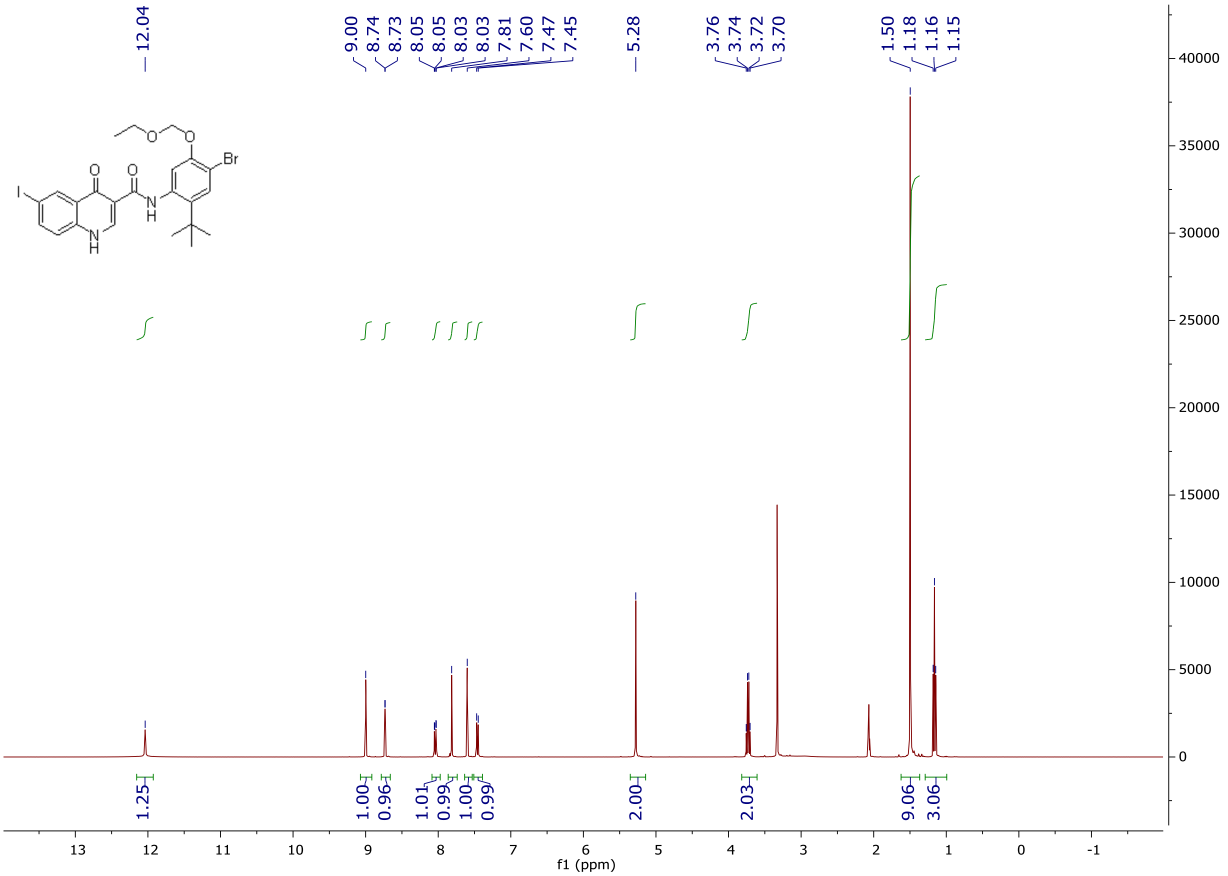

^{13}C NMR (Acetone-D_6):

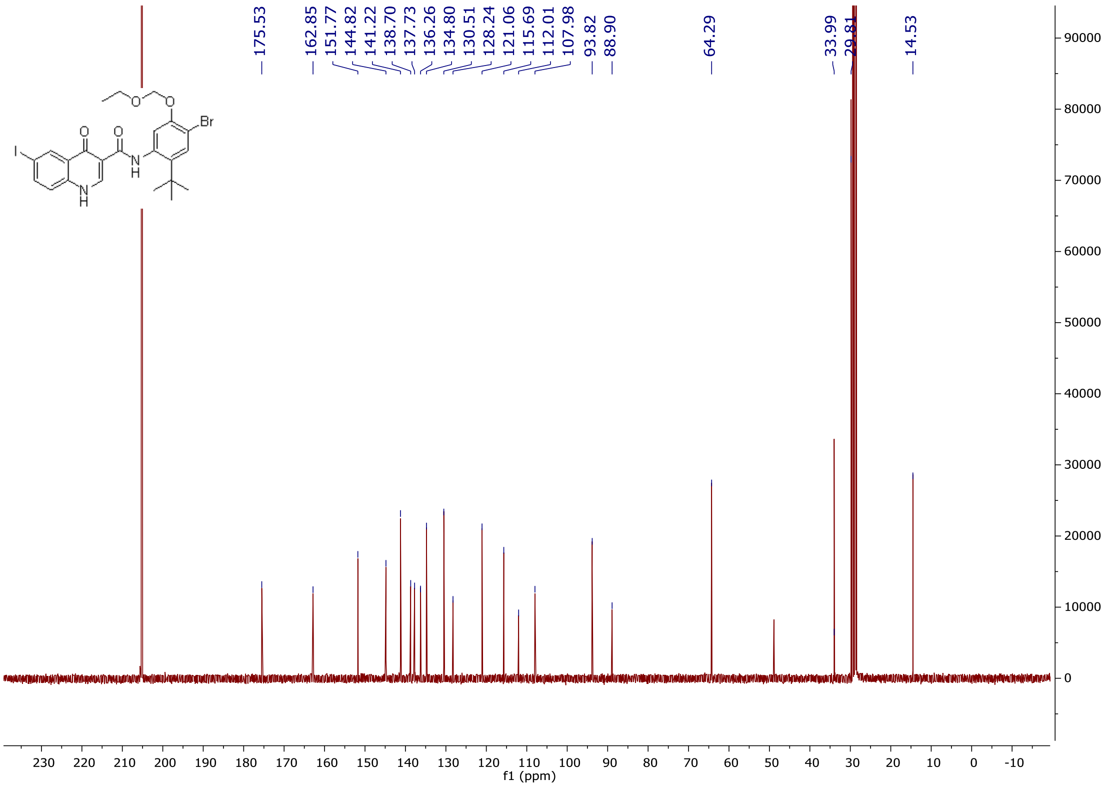

HRMS:

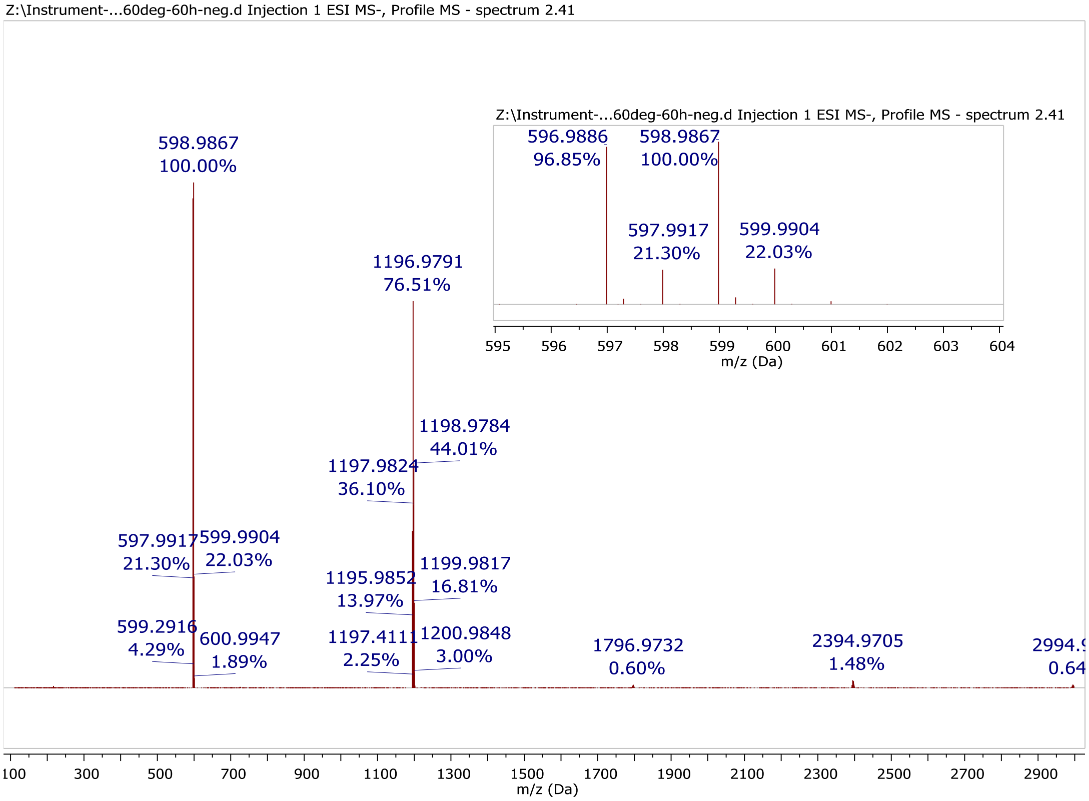

1.2. *N-(4-bromo-2-(tert-butyl)-5-(ethoxymethoxy)phenyl)-4-oxo-6-((trimethylsilyl)ethynyl)-1,4-dihydroquinoline-3-carboxamide (**7**)*

Chemical Formula: $C_{28}H_{33}BrN_2O_4Si$
Molecular Weight: 569.5710
Spectra provided: ^1H NMR , ^{13}C NMR

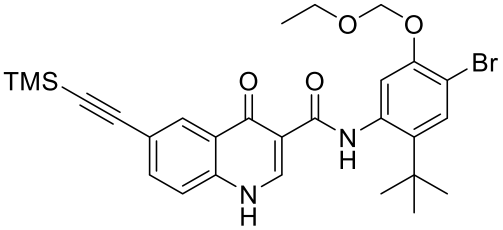

^1H NMR (Acetone-D_6):

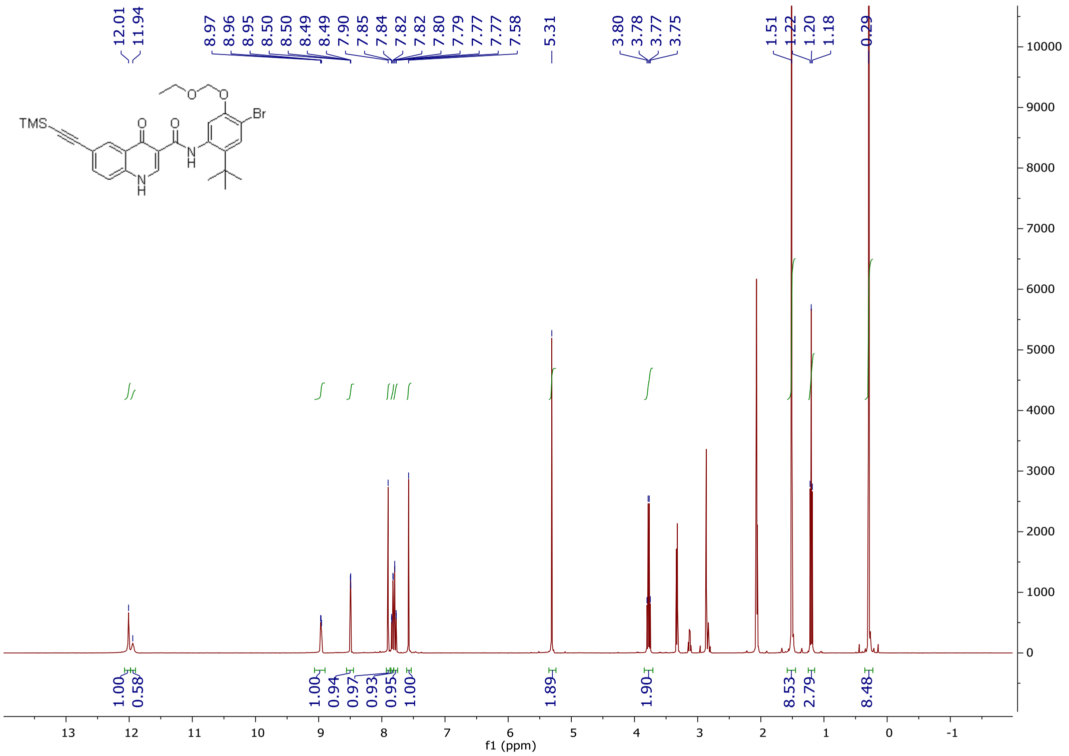

^{13}C NMR (Acetone-D_6):

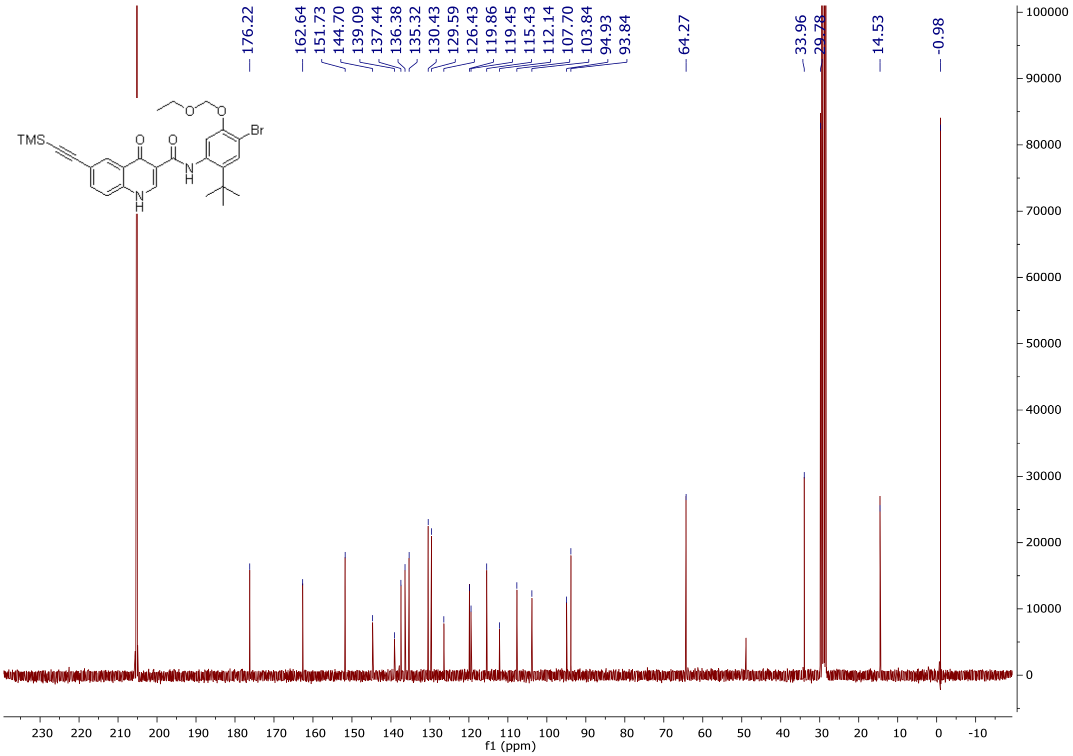

HRMS:

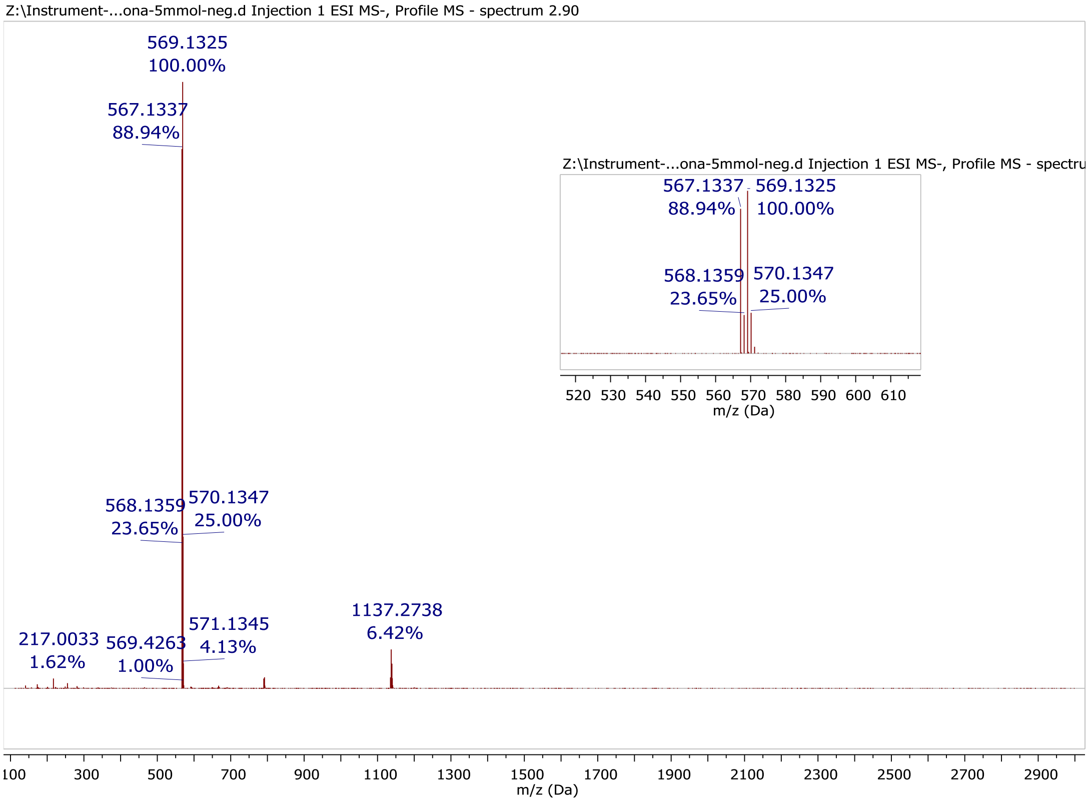

1.3. *N-(2-(tert-butyl)-5-(ethoxymethoxy)-4-(2,2,2-trifluoroacetyl)phenyl)-4-oxo-6-((trimethylsilyl)ethynyl)-1,4-dihydroquinoline-3-carboxamide* (**8**)

Chemical Formula: $C_{30}H_{33}F_3N_2O_5Si$
Molecular Weight: 586.6832
Spectra provided: HRMS

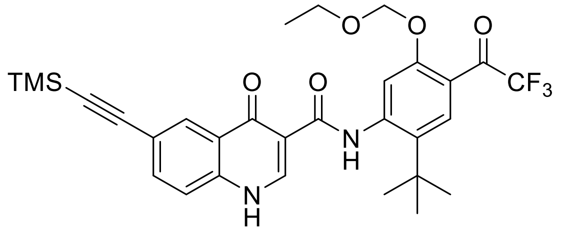

HRMS:

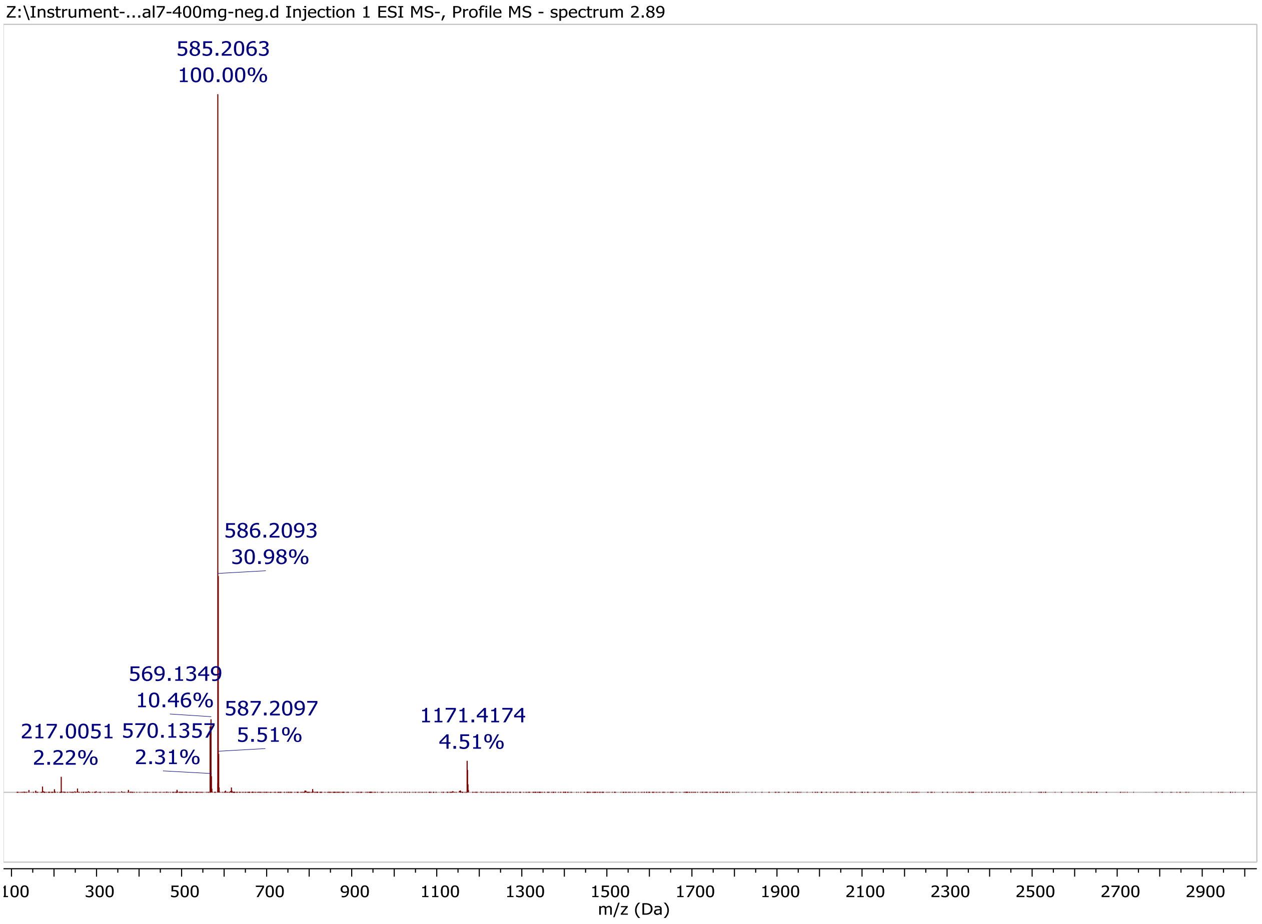

1.4. *NN-(2-(tert-butyl)-5-(ethoxymethoxy)-4-(2,2,2-trifluoro-1-(hydroxyimino)ethyl)phenyl)-4-oxo-6-((trimethylsilyl)ethynyl)-1,4-dihydroquinoline-3-carboxamide (9)*

Chemical Formula: $C_{30}H_{34}F_3N_3O_5Si$
Molecular Weight: 601.6982
Spectra provided: ^1H NMR , ^{19}F NMR, HRMS

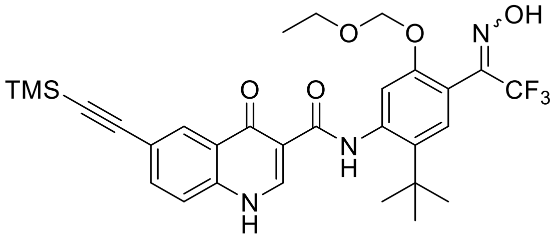

^1H NMR (Methanol-D_4):

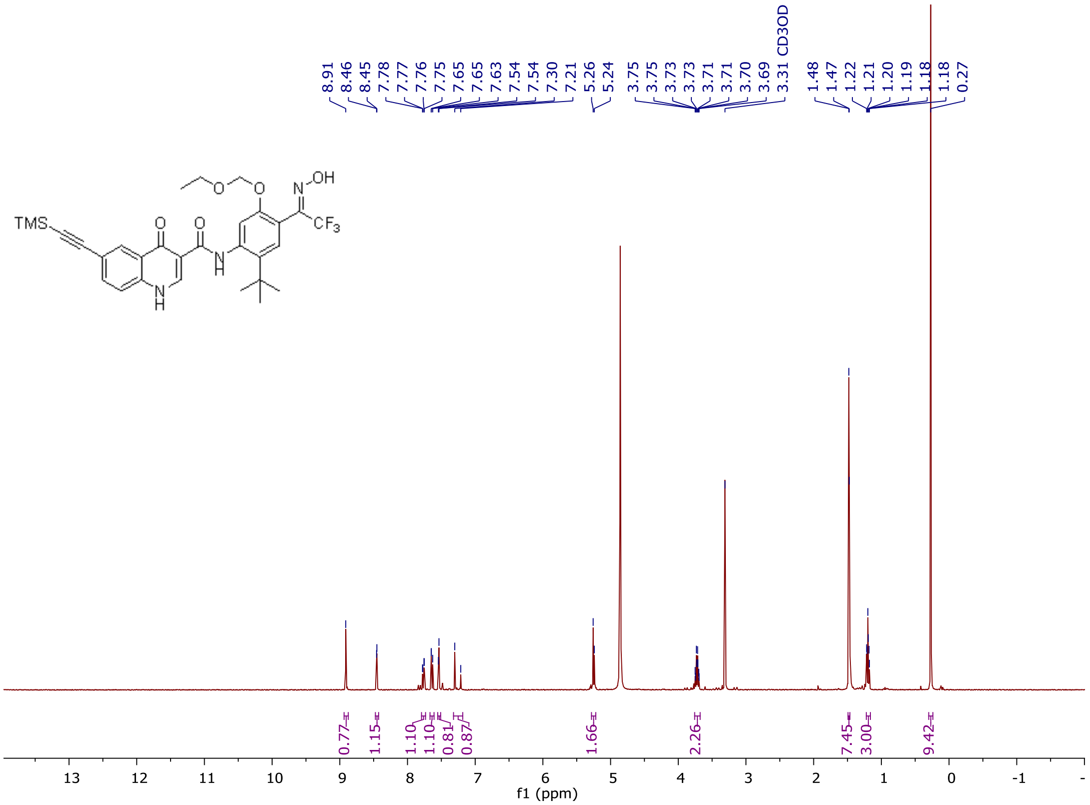

^{19}F NMR (Methanol-D_4):

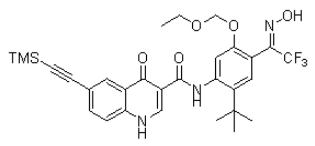

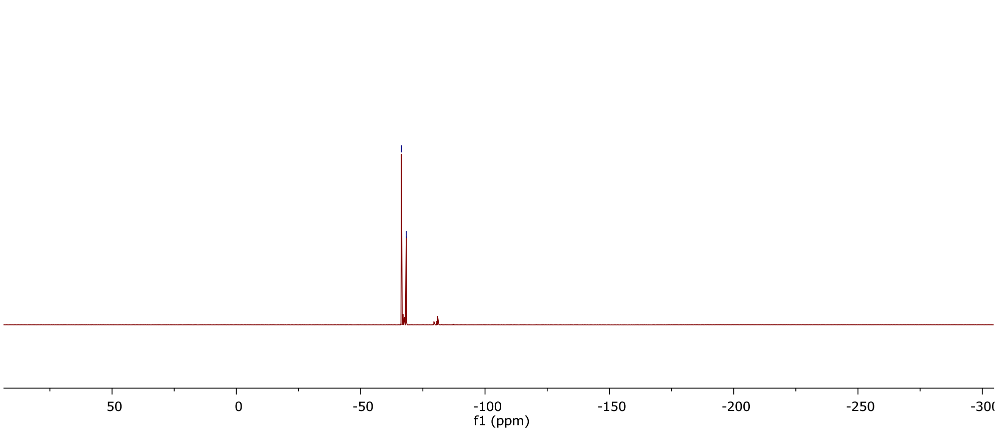

HRMS

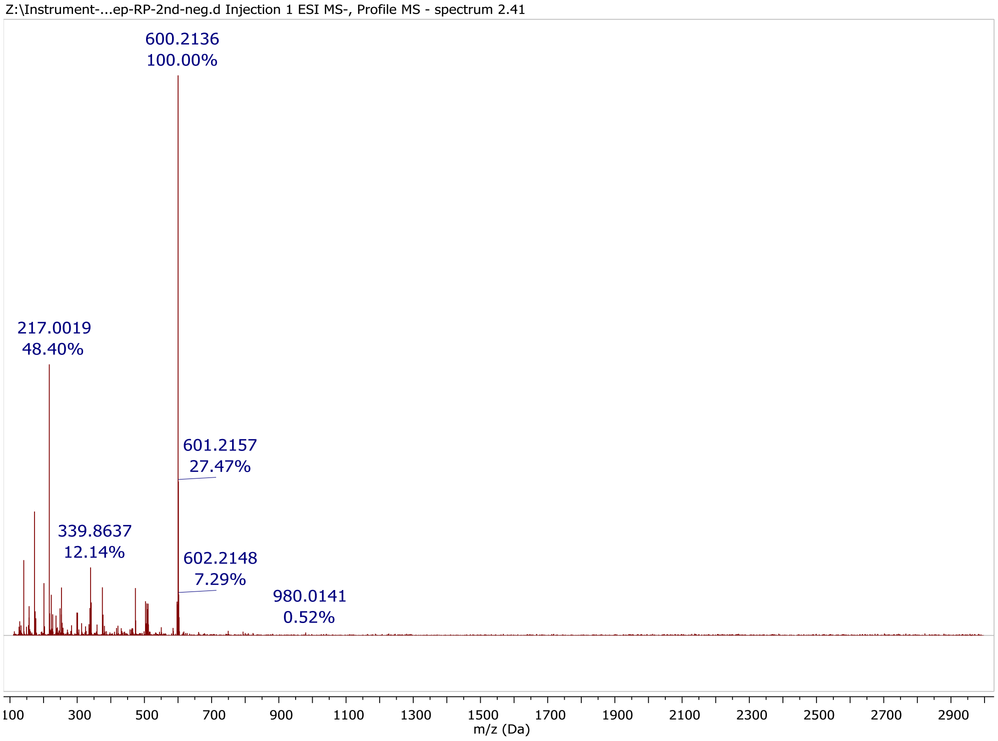

1.5. *N-(2-(tert-butyl)-5-(ethoxymethoxy)-4-(3-(trifluoromethyl)diaziridin-3-yl)phenyl)-6-ethynyl-4-oxo-1,4-dihydroquinoline-3-carboxamide (**10**)*

Chemical Formula: $C_{27}H_{27}F_3N_4O_4$

Molecular Weight: 528.5322

Spectra provided: ^1H NMR, ^{19}F NMR, ^{13}C NMR, HRMS

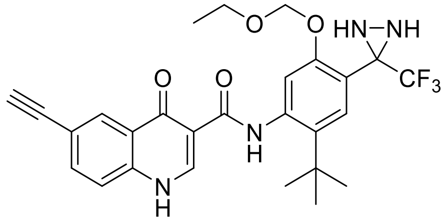

^1H NMR (Acetone-D_6):

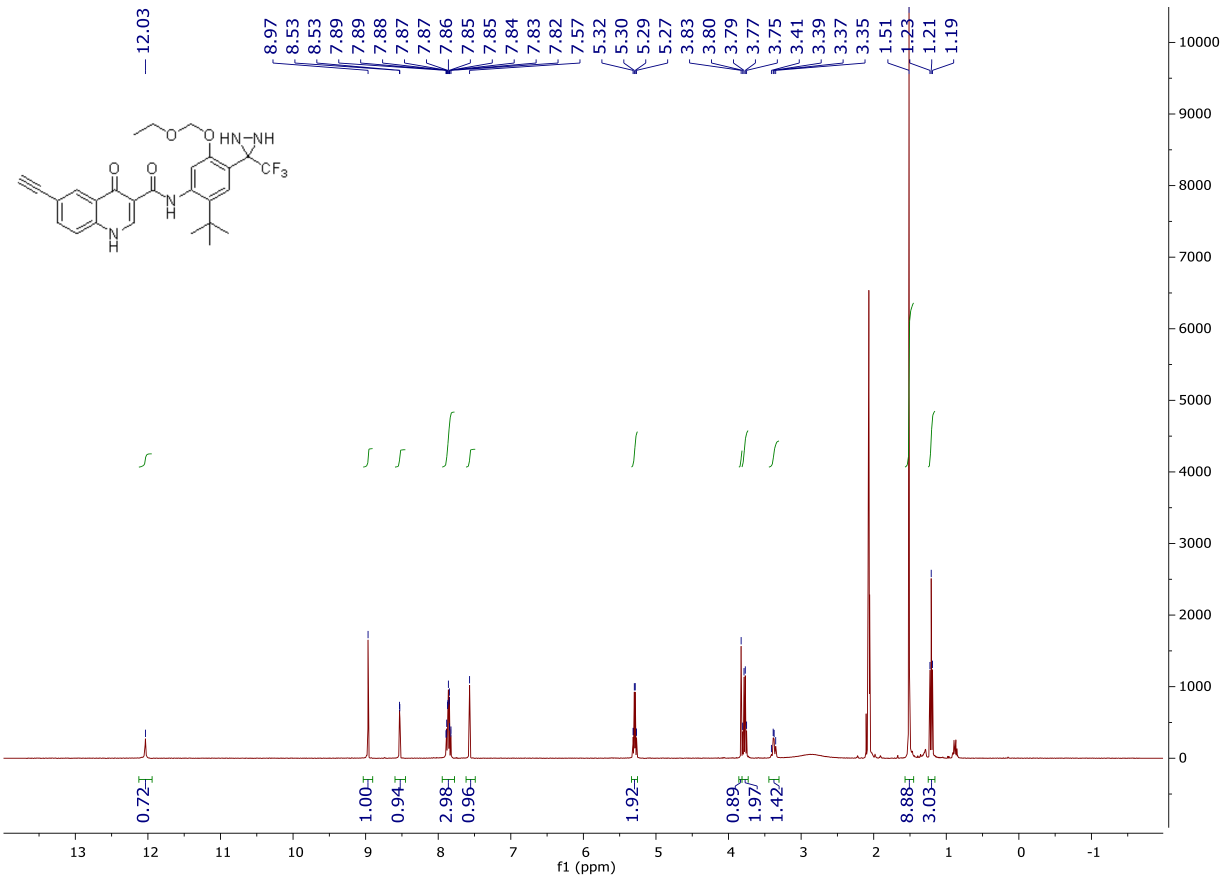

^{19}F NMR (Acetone-D_6):

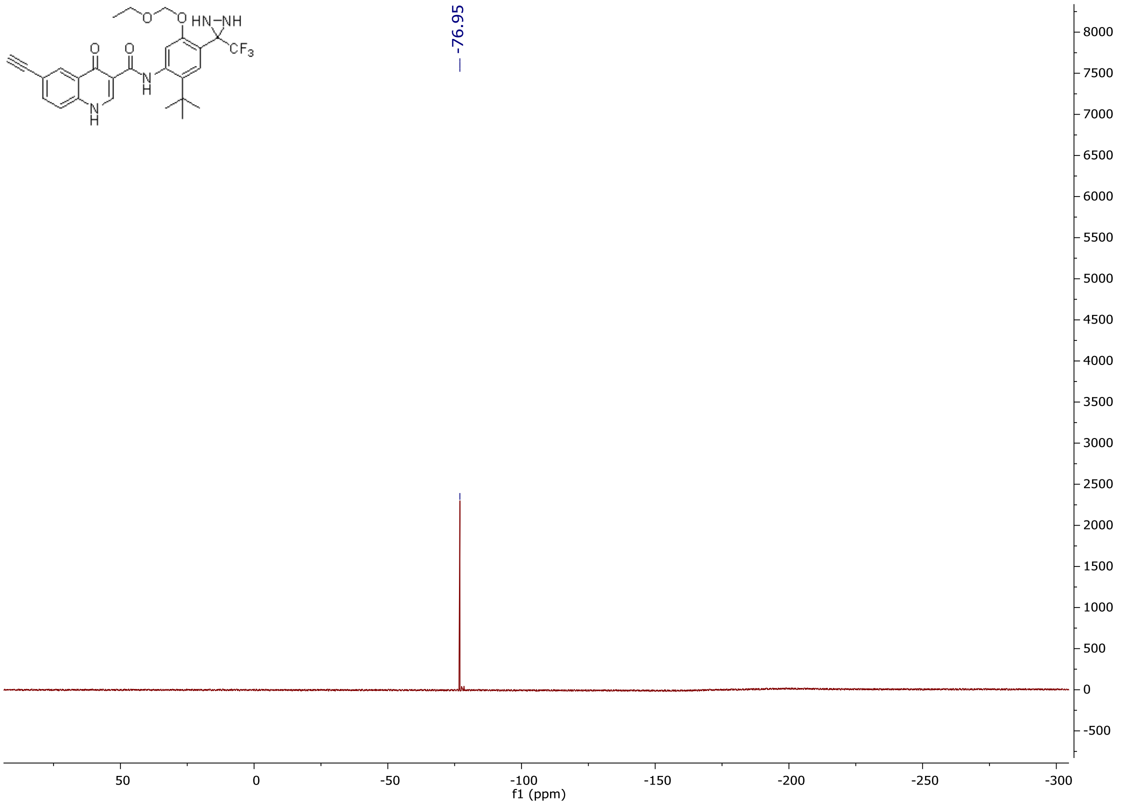

^{13}C NMR (Acetone-D_6):

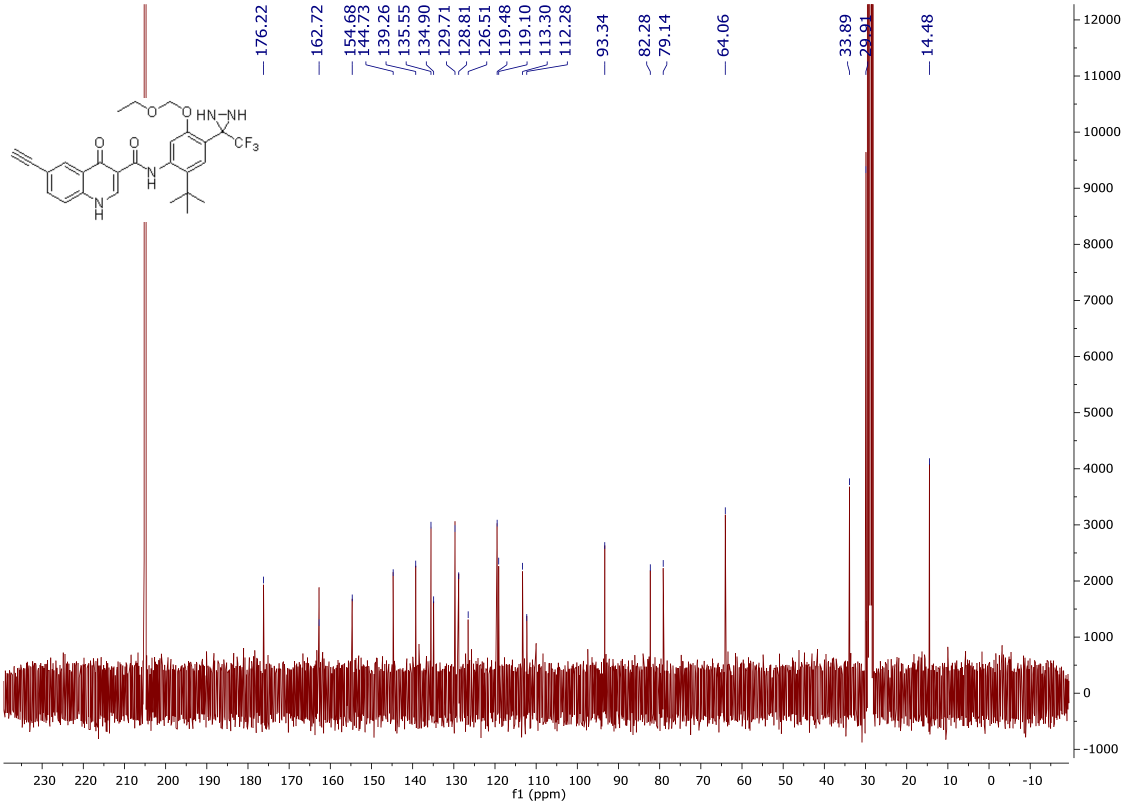

HRMS:

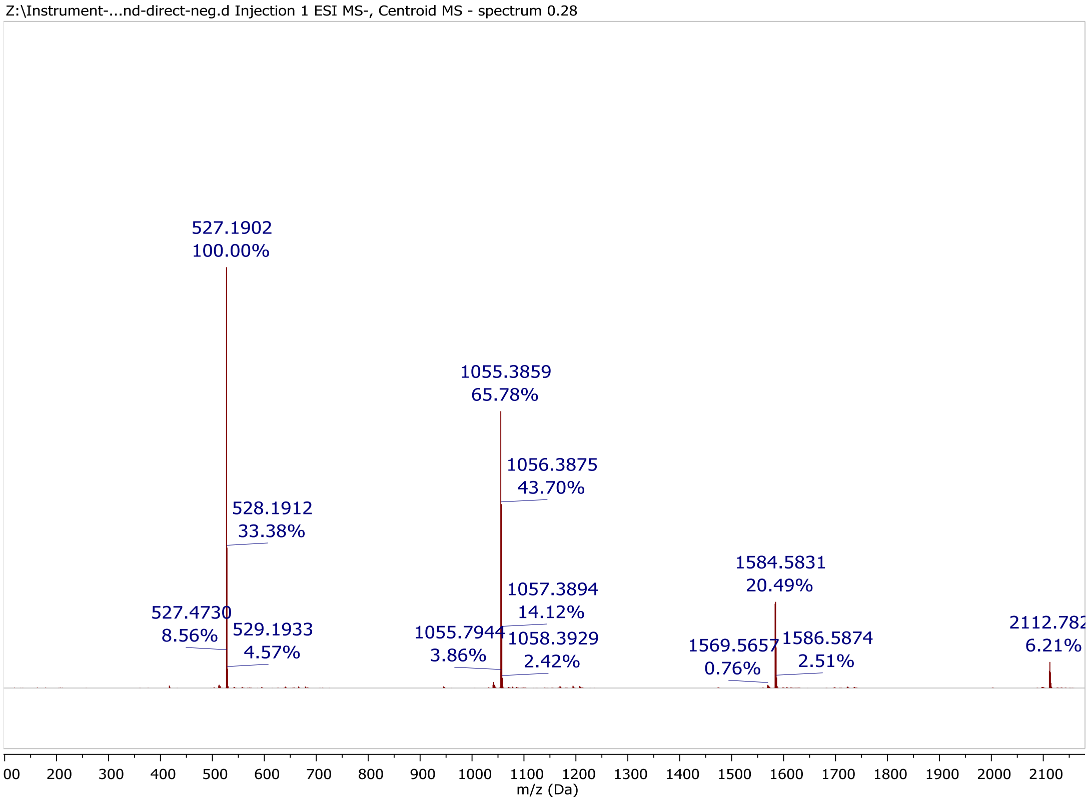
Z:\Instrument-...nd-direct-neg.d Injection 1 ESI MS-, Centroid MS - spectrum 0.28
527.1902
100.00%
1055.3859
65.78%
1056.3875
43.70%
528.1912
33.38%
1584.5831
20.49%
1057.3894
14.12%
527.4730
8.56%
529.1933
4.57%
1055.7944
3.86%
1058.3929
2.42%
1569.5657
0.76%
1586.5874
2.51%
6.21%
00
200
300
400
500
600
700
800
900
1000
1100
1200
1300
1400
1500
1600
1700
1800
1900
2000
2100
m/z (Da)

1.6. N-(2-(tert-butyl)-5-(ethoxymethoxy)-4-(3-(trifluoromethyl)-3H-diazirin-3-yl)phenyl)-4-oxo-6-(1-(13-oxo-17-((3aS,4S,6aR)-2-oxohexahydro-1H-thieno[3,4-d]imidazol-4-yl)-3,6,9-trioxa-12-azaheptadecyl)-1H-1,2,3-triazol-4-yl)-1,4-dihydroquinoline-3-carboxamide (**11**)

Chemical Formula: $C_{45}H_{57}F_3N_{10}O_9S$
Molecular Weight: 971.0672
Spectra provided: ^{1}H NMR, ^{19}F NMR, ^{13}C NMR, HRMS

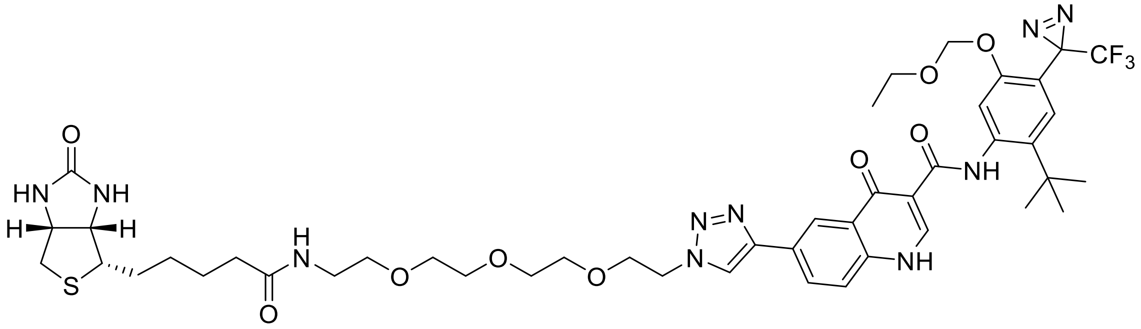

^{1}H NMR (Acetone-D_6):

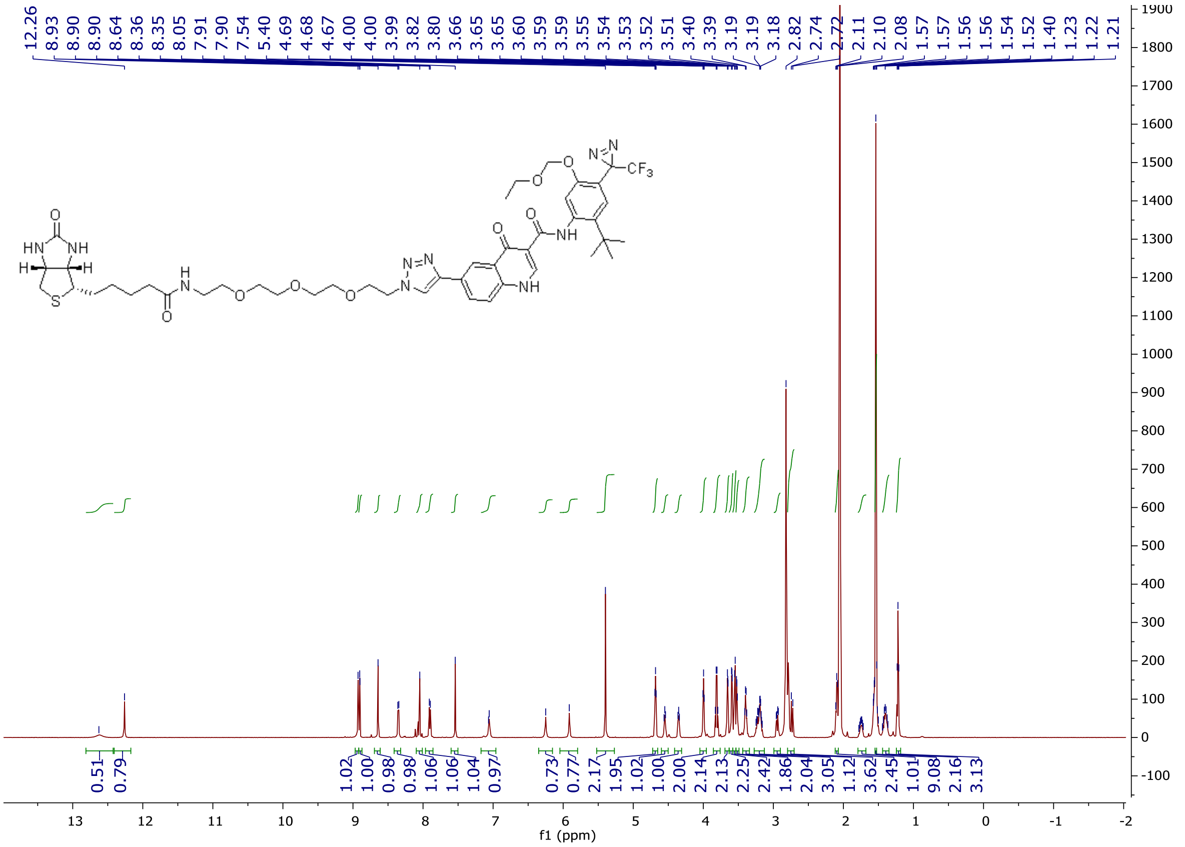

^{19}F NMR (Acetone-D$_6$):

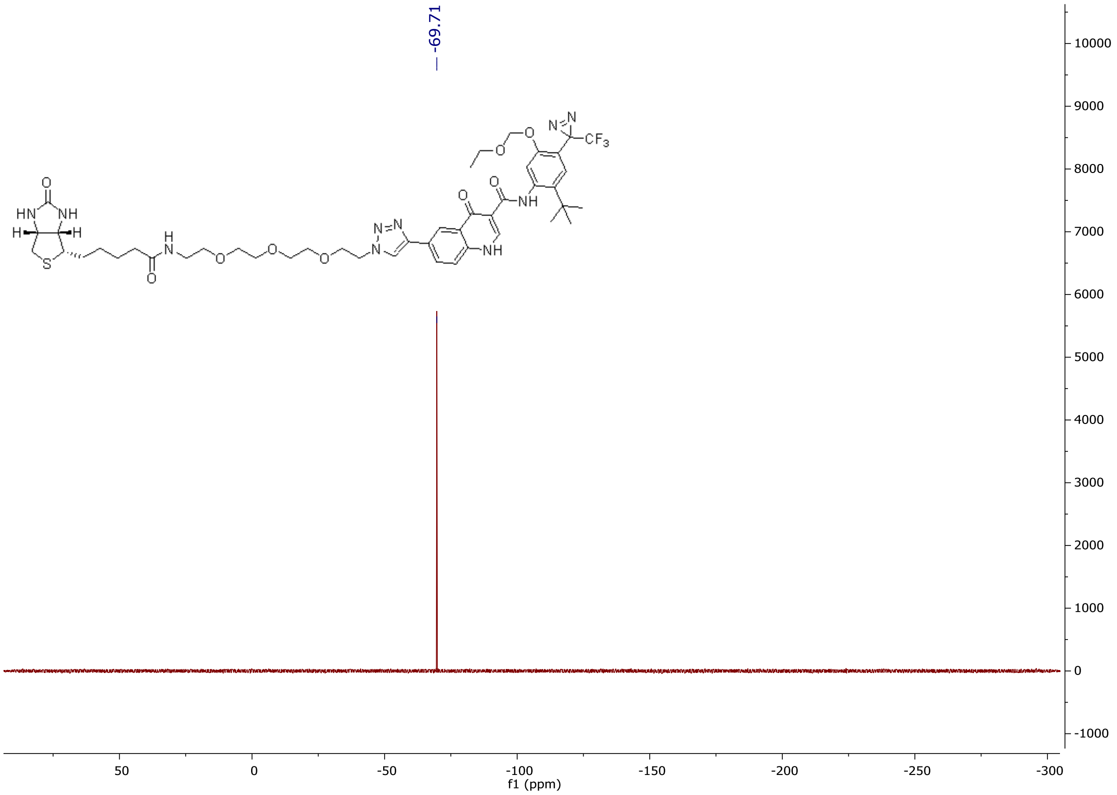

^{13}C NMR (Acetone-D$_6$):

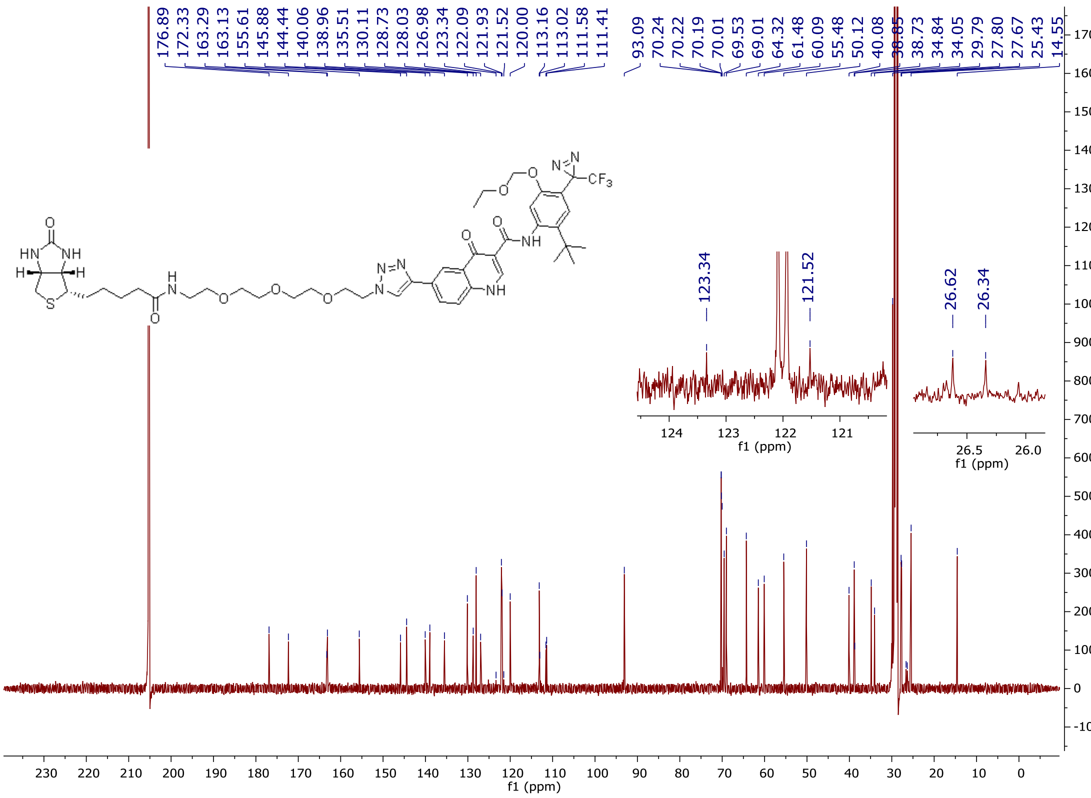

HRMS:

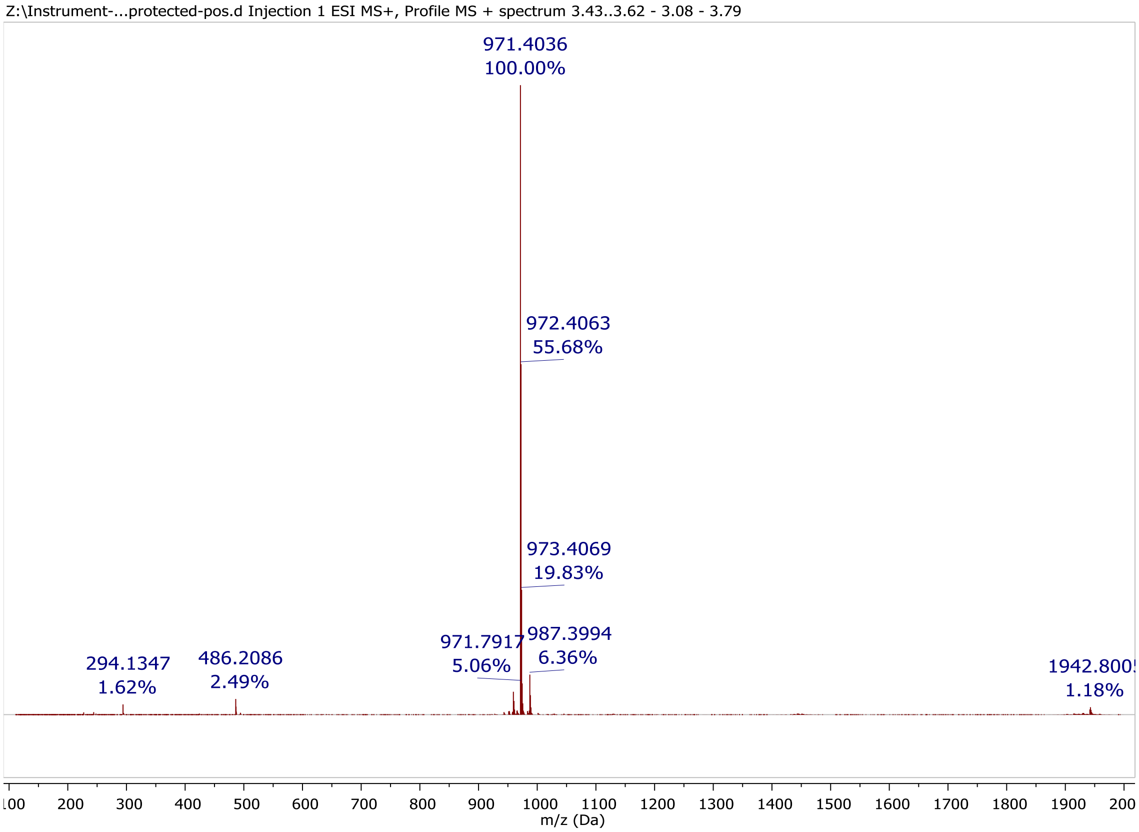

Z:\Instrument-...protected-pos.d Injection 1 ESI MS+, Profile MS + spectrum 3.43..3.62 - 3.08 - 3.79
971.4036
100.00%
972.4063
55.68%
973.4069
19.83%
971.7917
5.06%
987.3994
6.36%
294.1347
1.62%
486.2086
2.49%
1942.800
1.18%
100
200
300
400
500
600
700
800
900
1000
1100
1200
1300
1400
1500
1600
1700
1800
1900
200
m/z (Da)

1.7. *N-(2-(tert-butyl)-5-(ethoxymethoxy)-4-(3-(trifluoromethyl)-3H-diazirin-3-yl)phenyl)-4-oxo-6-(1-(13-oxo-17-((3aS,4S,6aR)-2-oxohexahydro-1H-thieno[3,4-d]imidazol-4-yl)-3,6,9-trioxa-12-azaheptadecyl)-1H-1,2,3-triazol-4-yl)-1,4-dihydroquinoline-3-carboxamide* (VX-770-BIOT, **3**)

Chemical Formula: $C_{42}H_{51}F_3N_{10}O_8S$
Molecular Weight: 912.9872
Spectra provided: ^{19}F NMR, HRMS, UV-Vis, 2-D Fluorescence spectra

The protected compound was treated with saturated HCl in MeOH at RT until complete deprotection, then the reaction was concentrated to give the final biotin diazirine which was stored as a DMSO solution at -80 °C in the dark. Quick decomposition was observed when attempting full characterization.

^{19}F NMR ($CDCl_3$):

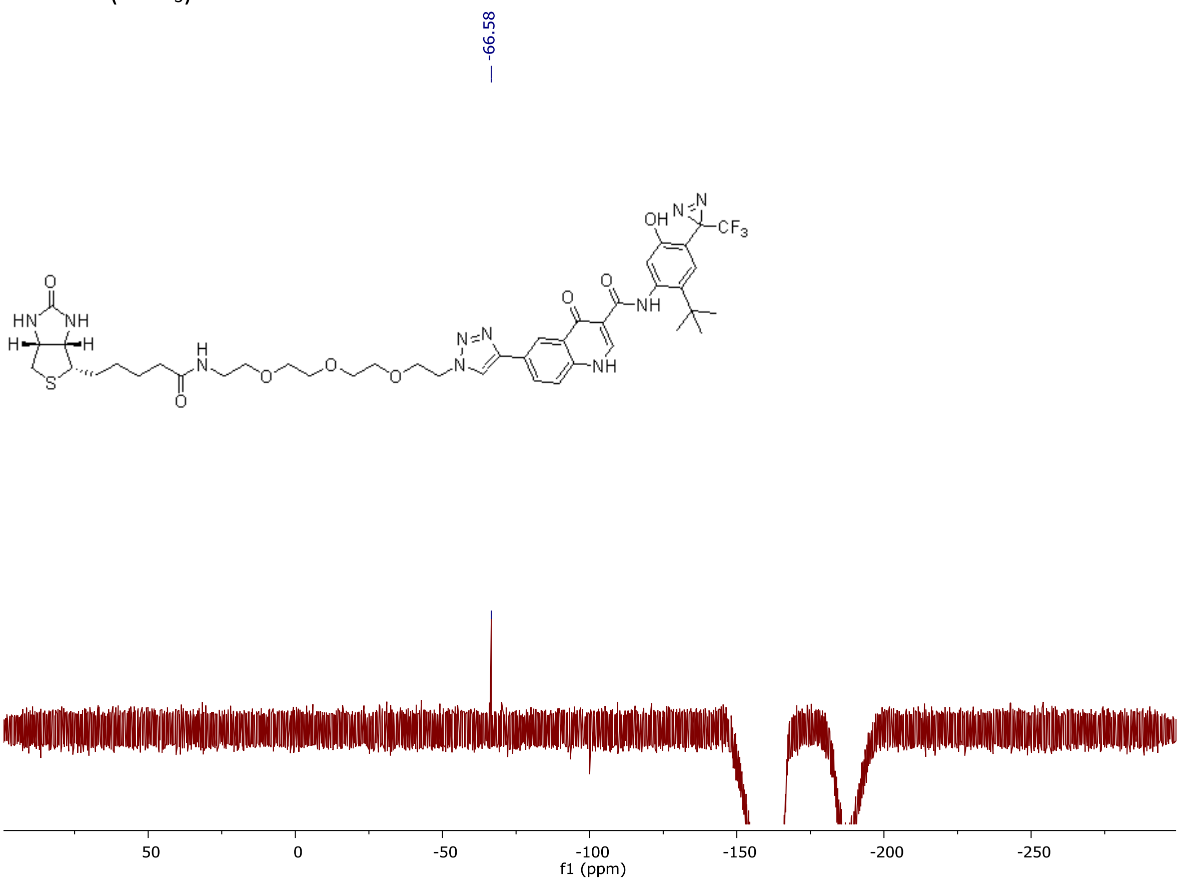

HRMS:

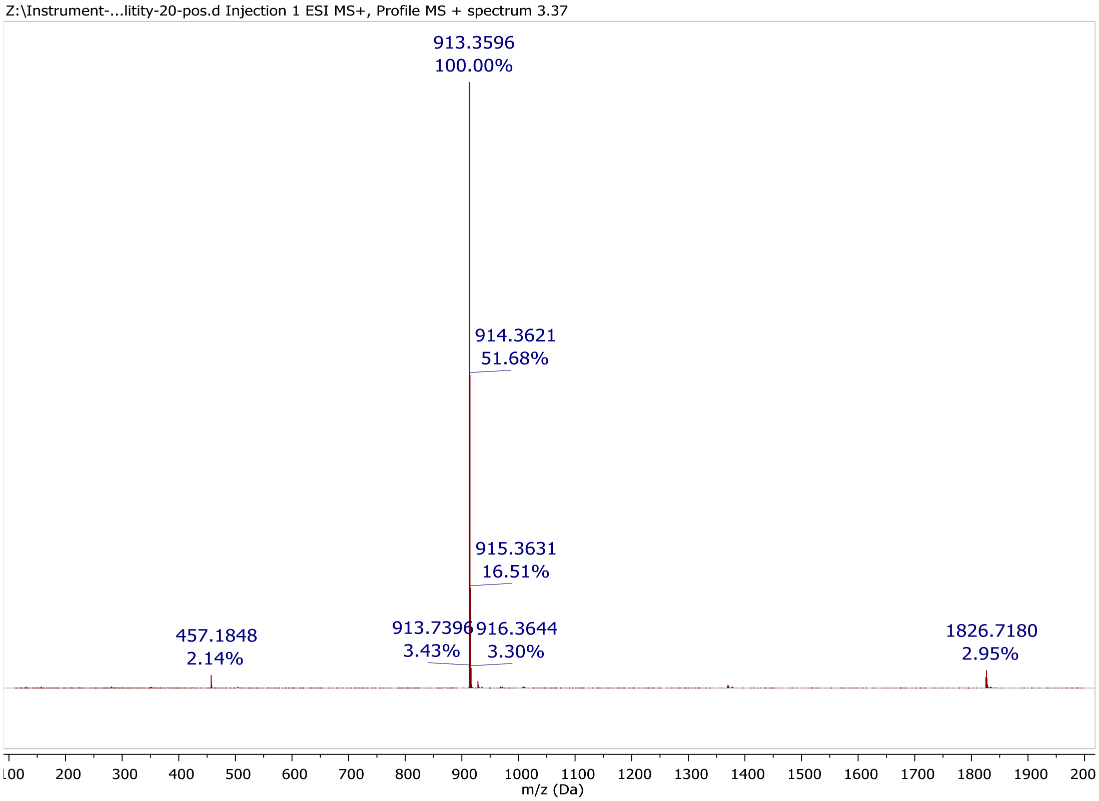

Z:\Instrument-...litity-20-pos.d Injection 1 ESI MS+, Profile MS + spectrum 3.37
913.3596
100.00%
914.3621
51.68%
915.3631
16.51%
457.1848
2.14%
913.7396
3.43%
916.3644
3.30%
1826.7180
2.95%
m/z (Da)

UV-Vis:

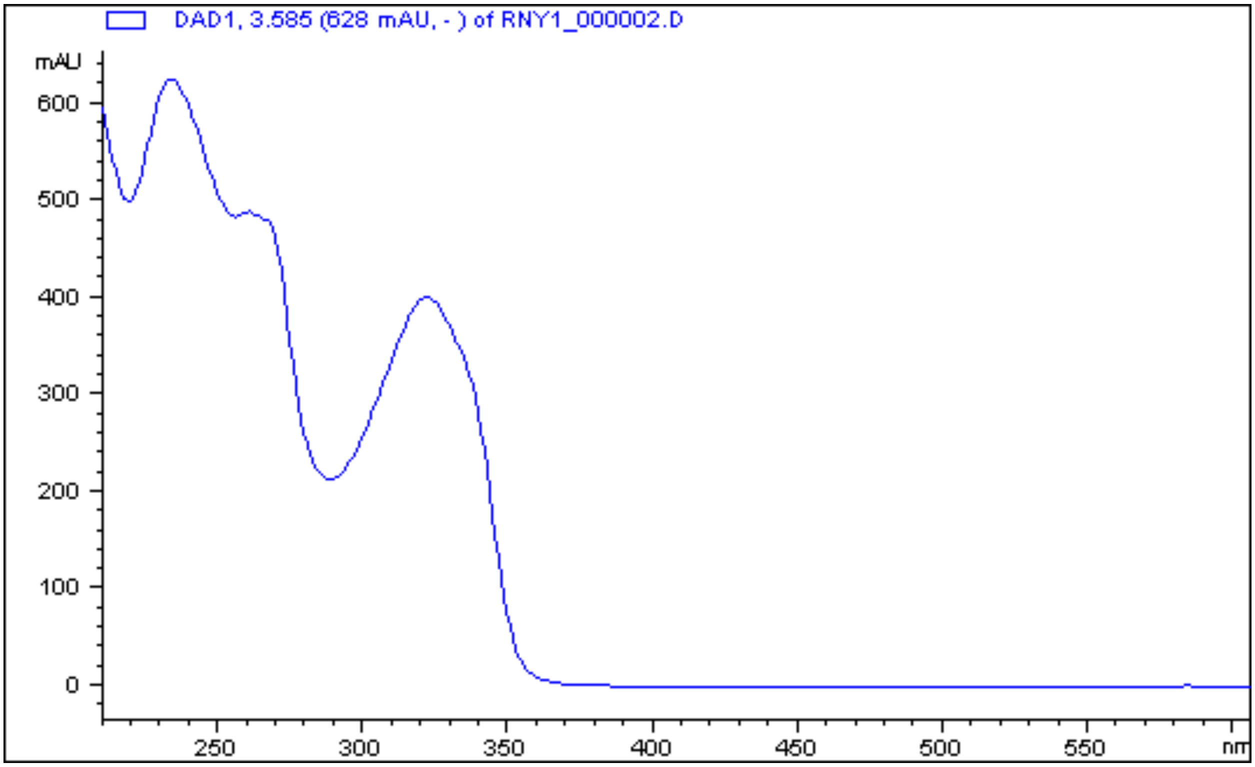

DAD1, 3.585 (628 mAU, -) of RNY1_000002.D
mAU
nm

2-D Fluorescence:

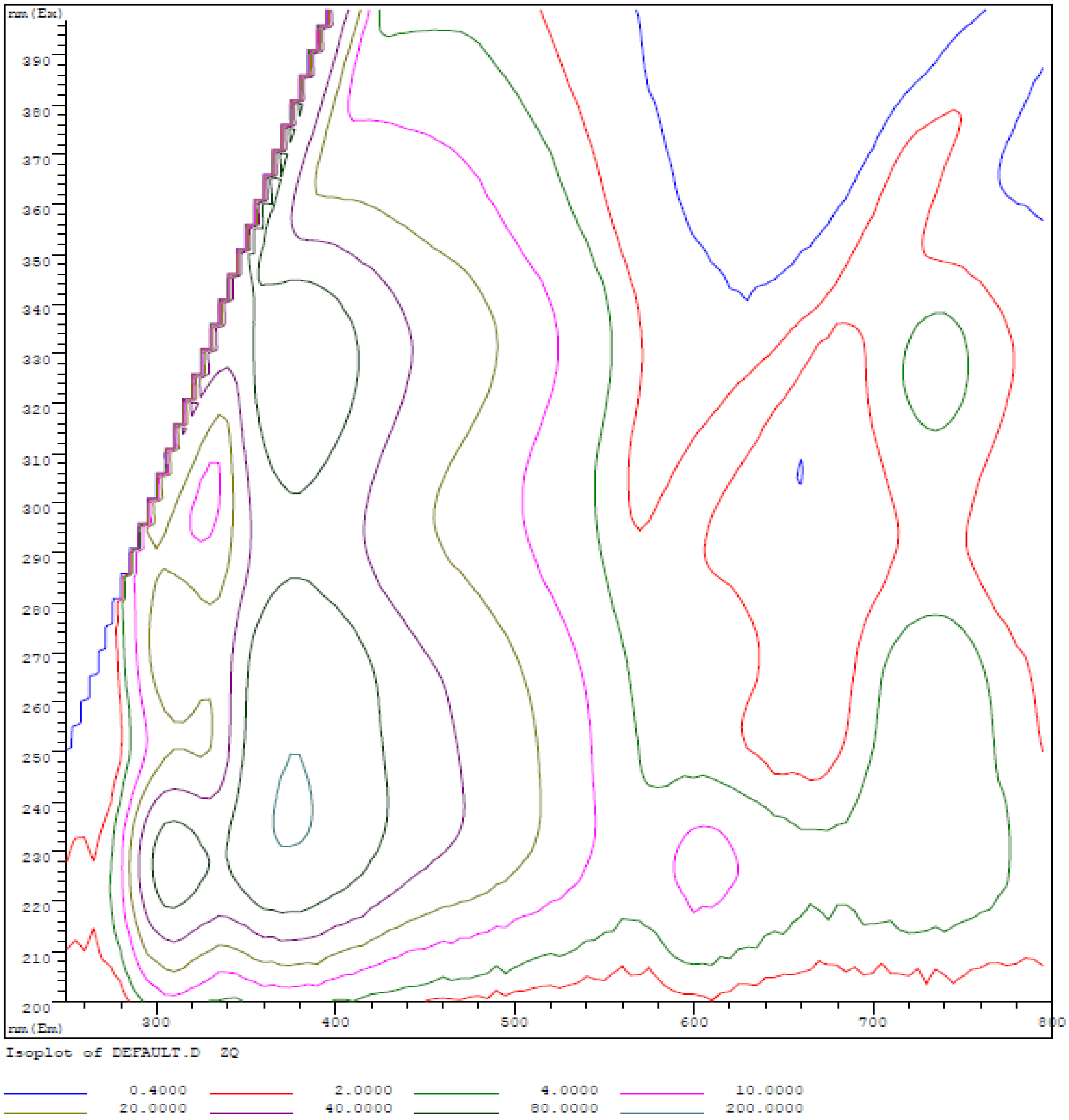

*Figure 2: 2D fluorescence spectrum of **3**, vertical axis: Excitation wavelength (nm), horizontal axis: emission wavelength (nm).*

2. Labeling of BSA with VX-770-BIOT (**3**)

Below are representative traces from the labeling of BSA with **3**. Conditions for labeling are as follows: BSA (1.5 nmol), **3** (7.5 mmol), 3% DMSO, 1 mL total volume in di.H_2O. BSA and probe were added to the solution, mixed and let to equilibrate for 30 minutes prior to exposure to 365 nm UV light. MS data was collected just after mixing (t = -30 min), after 30 minutes of equilibration (t = 0), and after 30 minutes of UV irradiation (t = 30 min). Traces below are from the t=0 and t=30 timepoints. For each timepoint spectra included are the A) the chromatograms of the Total Ion Current (TIC), with extracted ion current (EIC) for the masses of **3** (913/mz) and the resulting alcohol (903 m/z) that results from the reaction of the carbene with water; B) the +ESI scan showing the raw BSA signal; C) the deconvoluted mass spectra showing BSA (66430 amu) and the labeled-BSA (67315 amu).

In order to calculate the % labeling, the abundances of the signal for the labeled (67315 amu) and unlabeled (66430 amu) were used in the following formula:

% labeling = (abundance of labeled signal) / {(abundance of labeled + unlabeled signa) * 100

Timepoint t= -30 min

A)

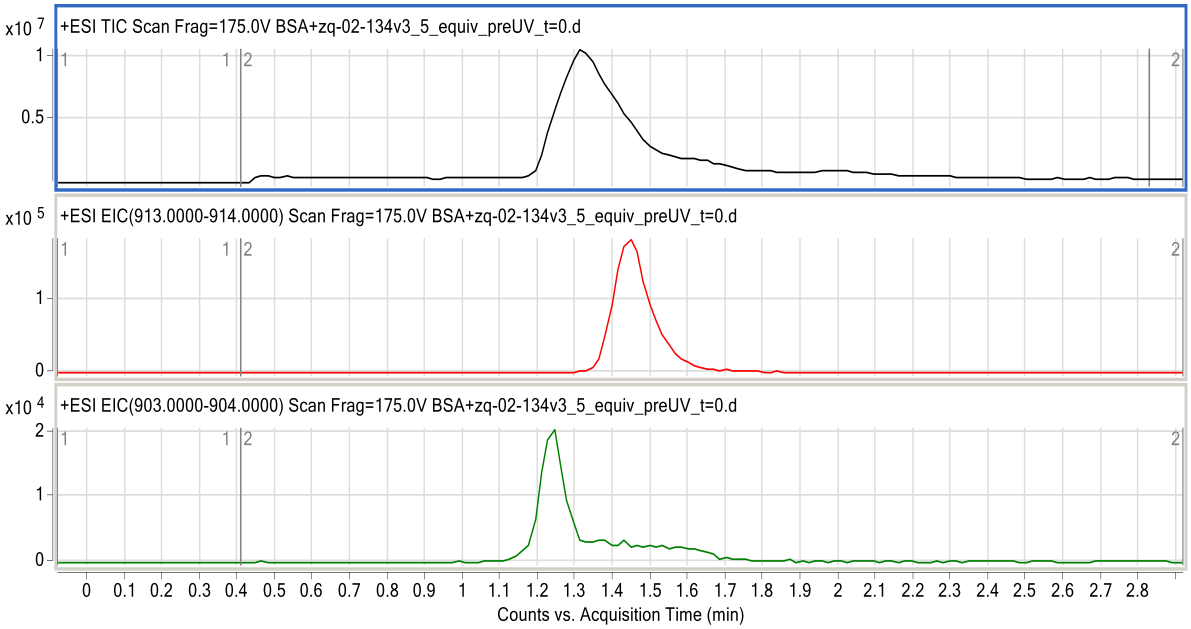

B)

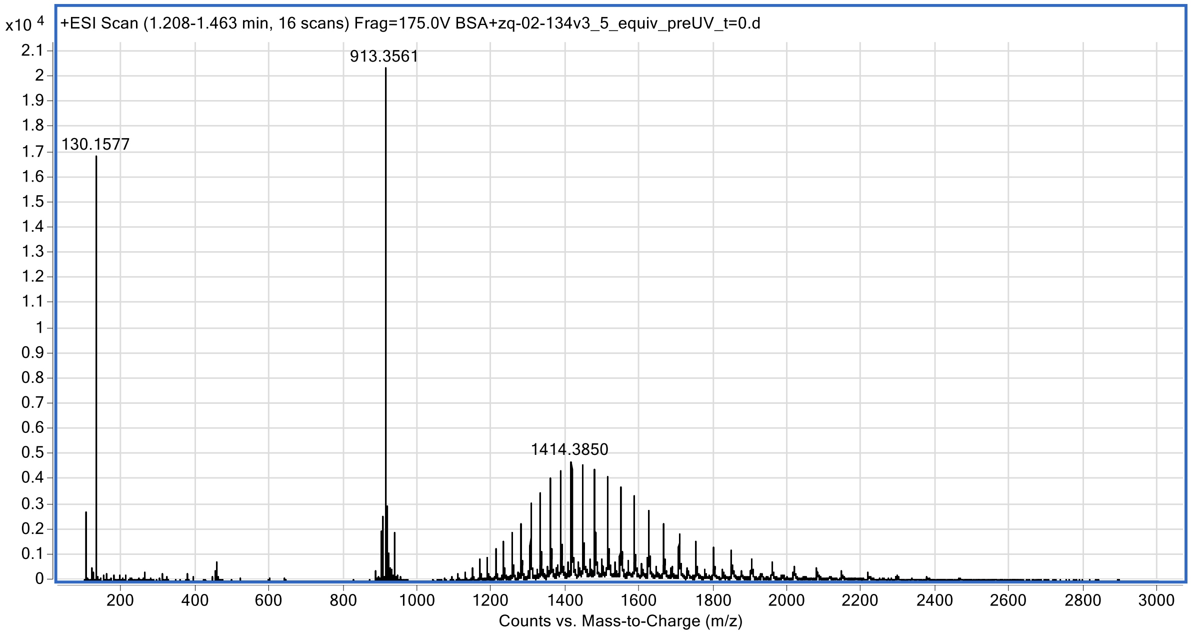

C)

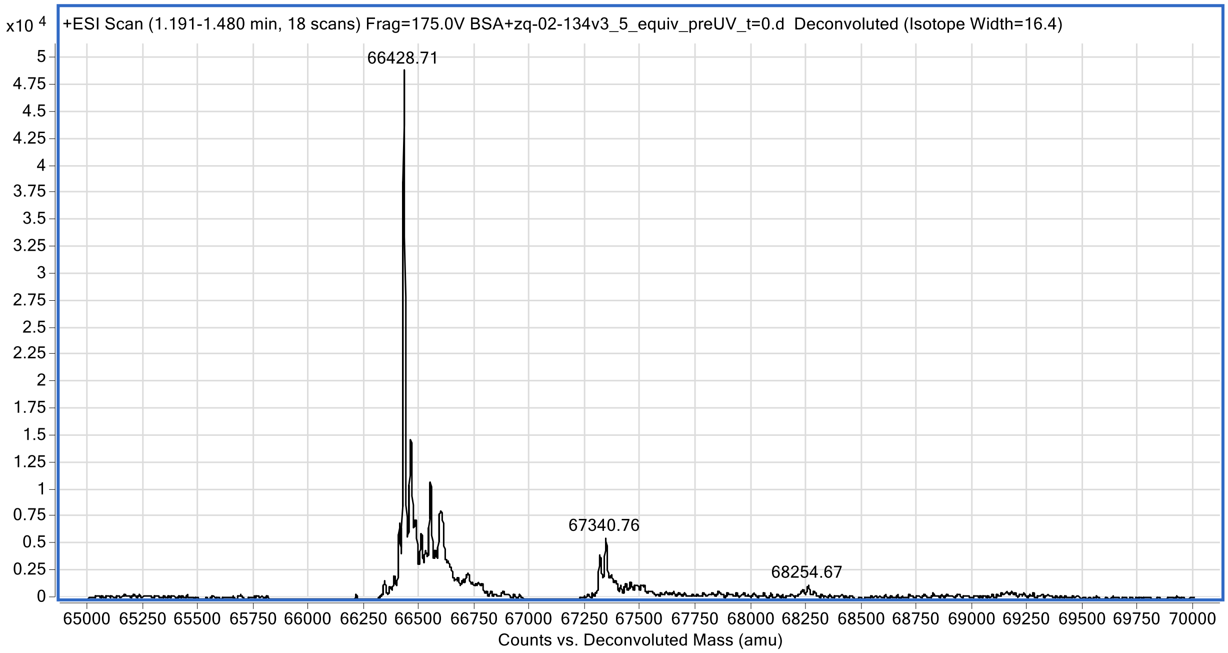

Mass (amu)	Abundance (count)
66428.72	48771
67315.11	4217
% labeling	8.0

Timepoint t = 0 min

A)

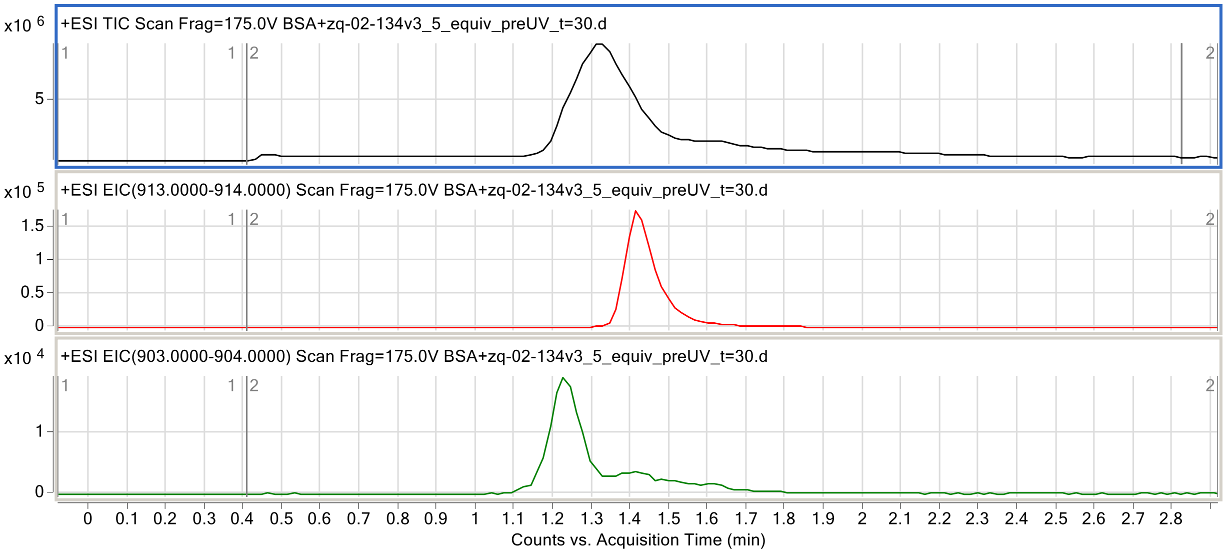

B)

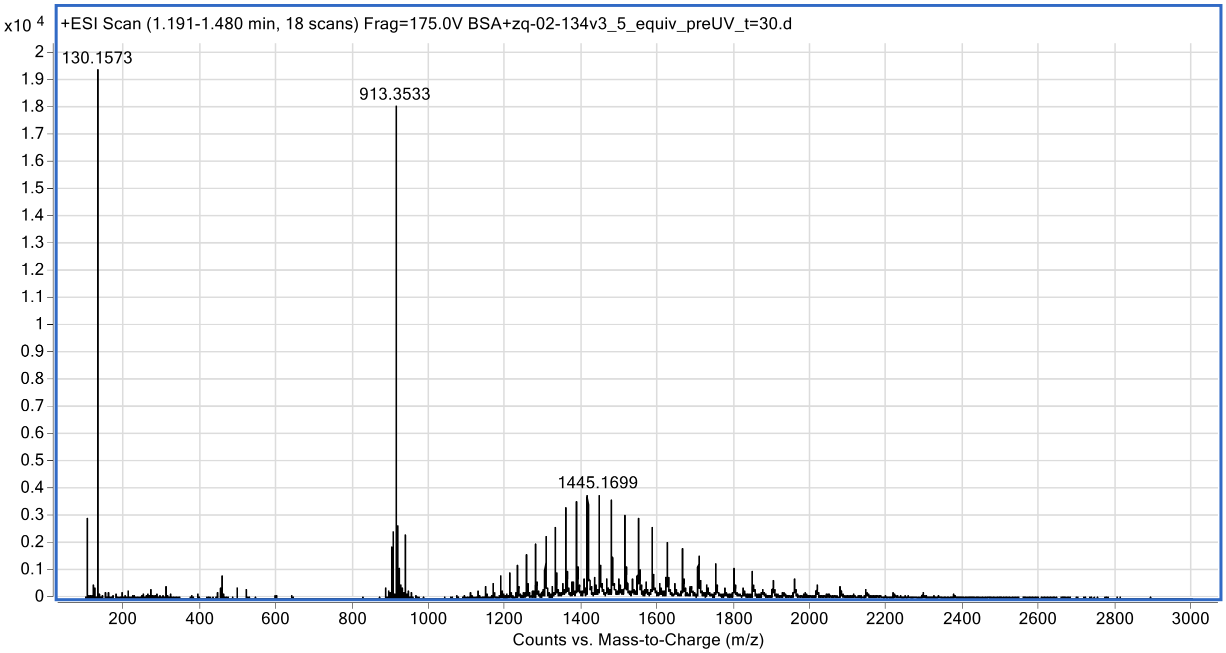

C)

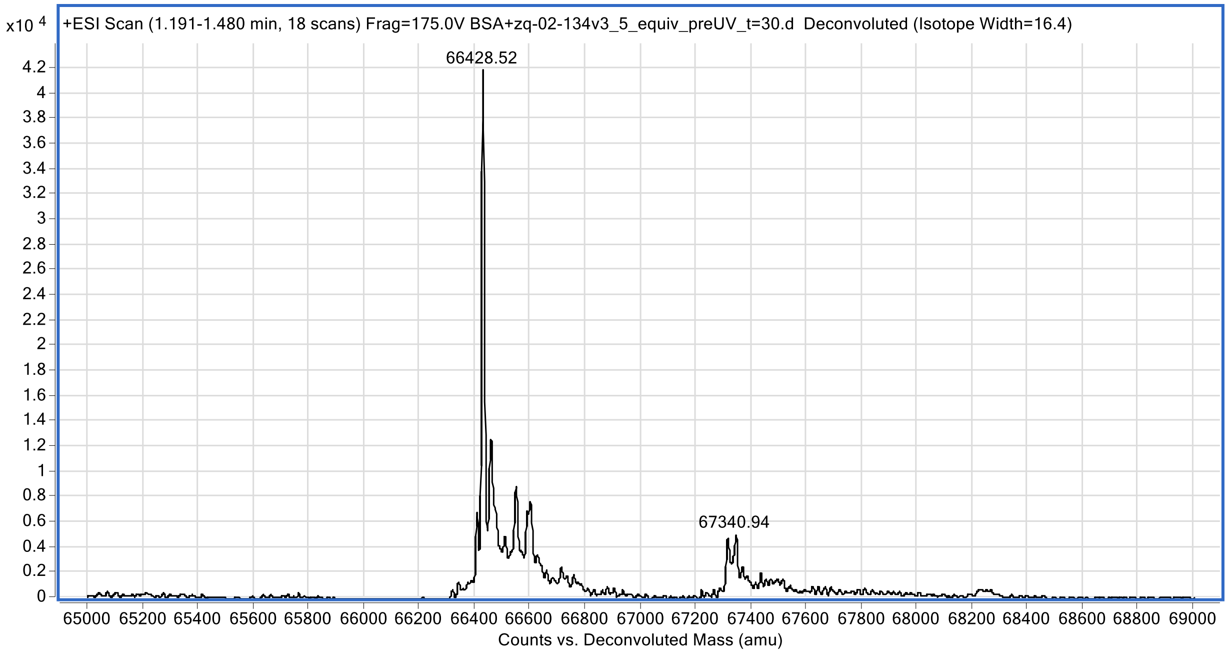

Mass (amu)	Abundance (count)
66428.52	41849
67314.79	4774
% labeling	10.2

Timepoint t = 30 min UV irradiation 365 nm

A)

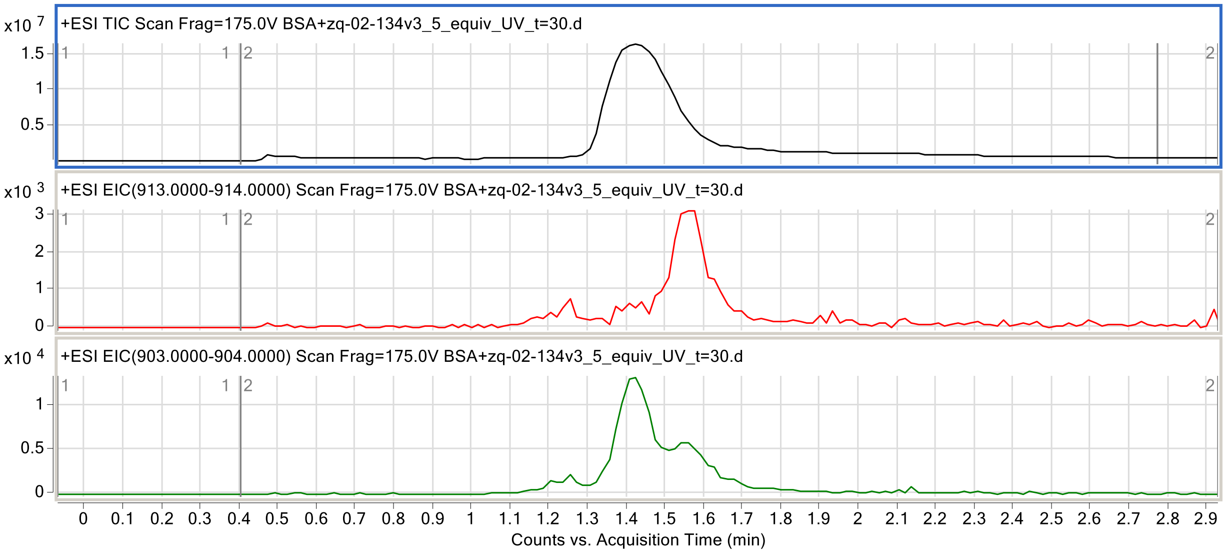

B)

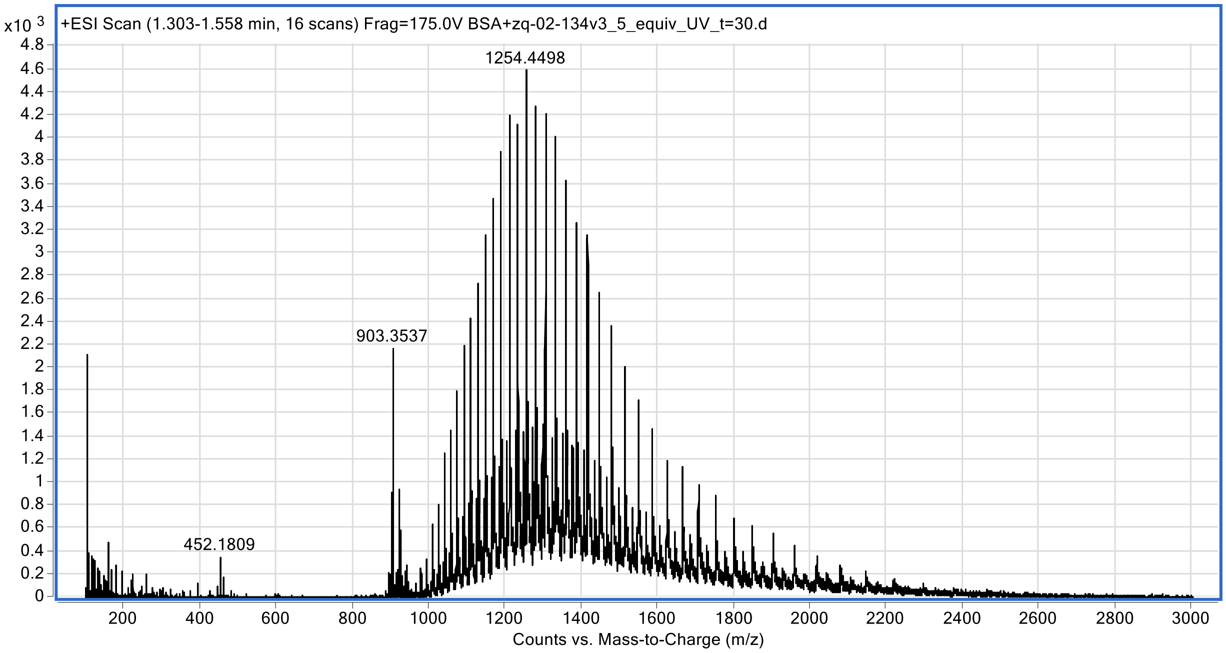

C)

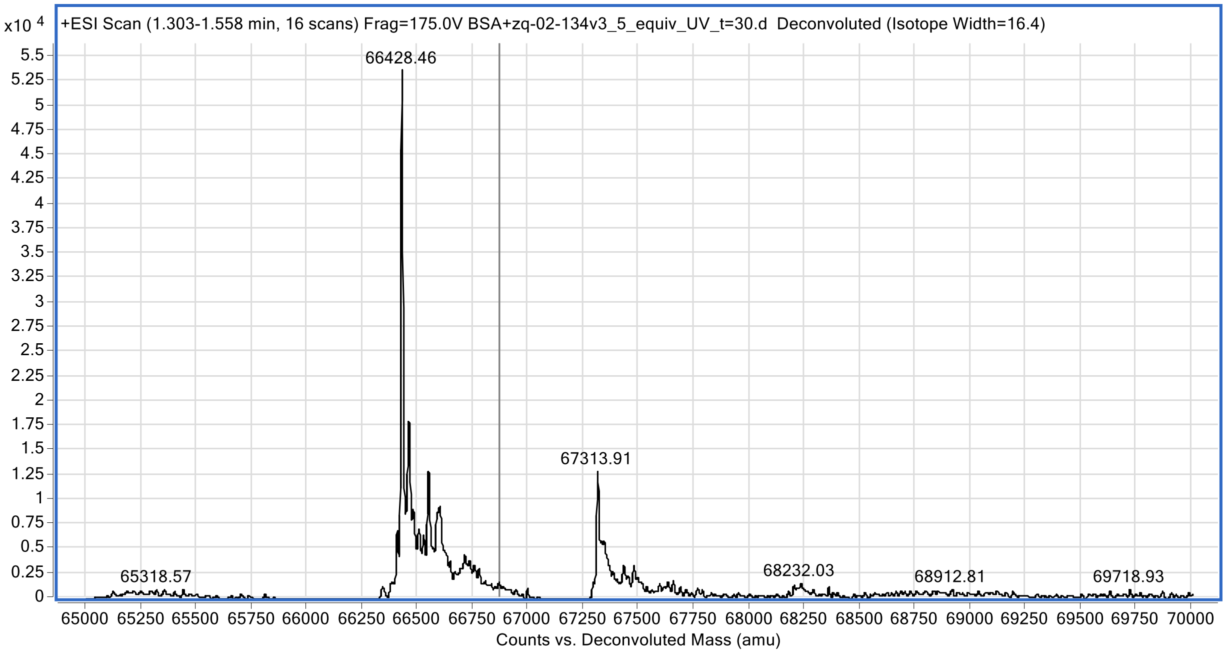

Mass (amu)	Abundance (counts)
66428.46	53551
67313.91	12970
% labeling	19.5
